# Development of Carbohydrate Polyelectrolyte Nanoparticles for Use in Drug Delivery Systems that Cross the Blood–Brain Barrier to Treat Brain Tumors

**DOI:** 10.3390/polym17121690

**Published:** 2025-06-18

**Authors:** Vladimir E. Silant’ev, Mikhail E. Shmelev, Andrei S. Belousov, Fedor O. Trukhin, Nadezhda E. Struppul, Aleksandra A. Patlay, Anna K. Kravchenko, Sergey P. Shchava, Vadim V. Kumeiko

**Affiliations:** 1 School of Medicine and Life Sciences, Far Eastern Federal University, Vladivostok 690922, Russia; shmelev.m.e@gmail.com (M.E.S.); belousov.ands@gmail.com (A.S.B.); fedortruhin2940@gmail.com (F.O.T.); struppul.nye@dvfu.ru (N.E.S.); patlay.al@mail.ru (A.A.P.); kravchenko.ak@dvfu.ru (A.K.K.); sergey_schava@yahoo.com (S.P.S.); 2 Laboratory of Electrochemical Processes, Institute of Chemistry, Far Eastern Branch of Russian Academy of Sciences, Vladivostok 690022, Russia; 3 A.V. Zhirmunsky National Scientific Center of Marine Biology, Far Eastern Branch of Russian Academy of Sciences, Vladivostok 690041, Russia

**Keywords:** nanocarrier, drug delivery systems, carbohydrates, polysaccharides, biomaterials, viscoelastic properties, blood–brain barrier, brain tumors, temozolomide

## Abstract

The low effectiveness of various brain cancer treatment methods is due to a number of significant challenges. Most of them are unable to penetrate the blood–brain barrier (BBB) when drugs are administered systemically through the bloodstream. Nanoscale particles play a special role among materials capable of binding drug molecules and successfully crossing the BBB. Biopolymeric nanoparticles (NPs) demonstrate excellent biocompatibility and have the remarkable ability to modify the environment surrounding tumor cells, thereby potentially improving cellular uptake of delivery agents. In our research, nanoscale polyelectrolyte complexes (PECs) ranging in size from 56 to 209 nm were synthesized by ionic interaction of the oppositely charged polysaccharides pectin and chitosan. The structural characteristics of these complexes were carefully characterized by infrared (FTIR) and Raman spectroscopy. The immobilization efficiency of antitumor drugs was comprehensively evaluated using UV spectrophotometry. The cytotoxicity of the NPs was evaluated in the U87-MG cell line. The preliminary data indicate a significant decrease in the metabolic activity of these tumor cells. Important details on the interaction of the NPs with an endothelial layer structurally similar to the BBB were obtained by simulating the BBB using a model based on human blood vessels. Our studies allowed us to establish a significant correlation between the kinetic parameters of drug immobilization and the ratio of biopolymer concentrations in the initial compositions, which provides valuable information for future optimization of drug delivery system design.

## 1. Introduction

The blood–brain barrier (BBB) is a highly selective protective barrier that, in combination with specific transport systems in microvascular endothelial cells, prevents the passage of most compounds of different molecular weights and polarities. The BBB is formed by specialized brain endothelial cells that continuously interact with surrounding cells such as astrocytes, pericytes, and perivascular macrophages, forming the so-called neurovascular unit [1].

The vascular network contributes to the transport of nutrients and oxygen to the brain and protects against potentially neurotoxic molecules. The functionality and organization of the BBB can be altered under pathological conditions, such as multiple sclerosis, epilepsy, autoimmune deficiency syndrome, dementia, stroke, and brain cancer [2]. This condition is commonly referred to as a blood–brain–tumor barrier. In low-grade gliomas, the normal vascularization and function of the blood–brain–tumor barrier remain mostly intact and resemble the BBB under normal conditions. However, high-grade gliomas are characterized by major alterations of normal vascular function, resulting in disruption [3].

Up to 98% of various drugs injected into the body both locally and systemically do not pass the BBB. This is the cause of high levels of disability and mortality from diseases of the brain and spinal cord [4,5,6]. Therefore, the lack of effective methods of drug delivery to the brain is a major obstacle to progress in therapy for neurological diseases, particularly brain tumors such as glioblastoma multiforme (GBM) [7,8].

GBM is one of the most aggressive primary brain tumors and is characterized by a negative prognosis for patients. The average life expectancy of patients after a standard course of treatment (surgery, radiotherapy, and chemotherapy) is approximately 15 months [9]. The main obstacle in combating this disease is the incomplete permeability of drugs through the BBB and the blood–tumor barrier formed, due to the disrupted blood network and high intracellular fluid pressure in the tumor area [10]. This impedes adequate penetration of antitumor agents to the affected areas, thereby decreasing the therapeutic effect [11].

Polyelectrolyte carbohydrate-based nanoscale particles are potential candidates as drug delivery systems against GBM due to their mimicking the brain’s extracellular matrix [12,13]. In addition, these materials are biocompatible and do not exhibit significant toxicity [14,15,16,17]. Induction of uptake of nanomaterials is faster if the nanoparticle (NP) composition is similar to the components of the tumor microenvironment. CD-44-mediated endocytosis is activated if hyaluronic acid is included in such delivery systems [18,19]. Furthermore, tuning of the nanomechanical properties of these NPs and actively targeting them to glioblastoma cell receptors using antibodies or aptamers further enhances the affinity of the nanomaterials for tumors [20]. Targeting NPs for CD133-dependent endocytosis, which transports cholesterol and lipoproteins from the extracellular space into glioblastoma cells, may also be an effective strategy for drug delivery to cells in this disease [21]. CD68-mediated phagocytosis is also common in glioblastoma cells and is induced when NPs are modified with lectins [22]. The integrin-dependent uptake pathway provides direct transfer of nanocarriers through E-cadherin-mediated interactions directly from endotheliocytes to glioblastoma cells [23]. NCAM-1, when overexpressed in glioma cells, plays an important role in neuronal and glial cell adhesion, and therefore can be used as a target for treatments aimed at glioma cells [24]. PD-L1 provides evasion of antitumor immunity due to its increased content and activity in glioblastoma cells. This may enhance the accumulation of structures on the surface of glioblastoma cells and cause further absorption of these structures into the cells [25].

The transport of molecules across the BBB is selective, depending on their molecular weight. Small molecules are usually delivered by simple diffusion (paracellular or transcellular) along a concentration gradient. Due to the presence of tight junctions, only a very small number of molecules can diffuse through the paracellular space, such as small lipophilic ones (<500 Da) [26]. Some hydrophilic or lipophilic molecules can be directly transported through endothelial cells [27]. Carriers are required to transport molecules with high weight and polarity. Transport can be categorized as facilitated diffusion (along a concentration gradient) or active transport using adenosine triphosphate against a concentration gradient. Some examples of facilitated diffusion are the carriers of hexoses, amino acids, monocarboxylic acids, or fatty acids using glucose transporter isoform 1 GLUT1/SLC2A1, large neutral amino acid transporter 1 LAT1/SLC7A5, or monocarboxylic acid transporter/MCT1 [28]. Some mechanisms for overcoming barriers through NPs are summarized in the articles listed in Table S1 [18,20,23,24,25,29,30,31,32,33,34,35].

In this work we obtained NPs based on two oppositely charged polyelectrolytes—chitosan and pectin. The surface of the NPs was modified with fluorescent molecules and antibodies specific to brain tumor cells (gliomas) for their subsequent tracking in vitro experiments and targeting. The possibility of passing the obtained drug delivery systems through a BBB model was investigated. Possible variants of drug immobilization in NPs were studied and modeled using temozolomide (TMZ) as an example.

## 2. Materials and Methods

### 2.1. Materials

High molecular weight chitosan (molecular mass 200 kDa, degree of deacetylation ~80%, JSC “Bioprogress”, Moscow, Shchelkovo, Russia), glacial acetic acid (98%, “VECTON”, Saint-Petersburg, Russia), pectin (molecular mass 120 kDa, degree of esterification ~10%, low-esterified pectin was obtained from commercial pectin (Herbstreith & Fox GmbH & Co Group, Neuenbürg, Germany) by using the acid method [7]), 2-[4-[4-(2-hydroxyethyl)piperazin-1-yl]ethane-1-sulfonic acid (HEPES) (99.5%, Suzhou Yacoo 2Science Co., Suzhou, China), sodium chloride (99.5%, Sigma Aldrich, Taufkirchen, Germany), TMZ, which is the most commonly used drug in glioma treatment (Temomid, lyophilisate for preparation of injection solution, Jodas Expoim Ltd., Telangana, India), dimethyl sulfoxide (DMSO) (purity 100%, Cisco Ltd., Sriperumbudur, India), potassium hydrophosphate (99%, Molecula Ltd., Darlington, UK), potassium dihydrophosphate (99%, Amresco, Solon, OH, USA), primary antibody to NCAM1 (neuronal adhesion molecule) (Host-rabbit, LOT-DF7832, Affinity, Jiangsu province, China), primary antibody to PDL1 (programmed death ligand) (Host-rabbit, LOT-PAA788Hu01, Affinity, Jiangsu province, China), Alexa-Fluor 568 secondary antibody (Host-rabbit, LOT-A11036, Affinity, Jiangsu province, China), Tween-20 (99.5%, Suzhou Yacoo Science Co, Suzhou, China), fluorescein isothiocyanate (FITC) (Cisco Ltd., Sriperumbudur, India), 1-[3-(dimethylamino)propyl]-3-ethylcarbodiimide hydrochloride (EDC) (97%, Srichem, Shanghai, China), N-hydroxysuccinimide (NHS) (99%, Srichem, Shanghai, China), Rhodamine 6G (BHA, LenReactiv, Saint-Petersburg, Russia), Hoechst 33342 triglyceride (Sigma Aldrich, St. Louis, MO, USA), paraformaldehyde (96%, extrapure, Acros organics, Belgium, WI, USA), sodium azide (99.7%, extrapure, Amresco, Solon, OH, USA), Triton X-100 (99.5%, Suzhou Yacoo Science Co., Suzhou, China), sodium pyruvate (NeoFroxx, Einhausen, Germany), fetal bovine serum (Cell Technologies, Vancouver, BC, Canada), DMEM nutrient medium (Capricorn Scientific, Ebsdorfergrund, Germany), Locktide 401 superglue (Henkel, Rocky Hill, CT, USA), and phosphate-buffered saline (PBS) (Sigma Aldrich, St. Louis, MO, USA) were purchased. The deionized water used in the experiments was obtained using a Milli-Q IQ-7000 water purification system (Merck Millipore, Rahway, NJ, USA).

### 2.2. Nanoparticle Preparation

Polyelectrolyte-based NPs were prepared by ionic gelation. A 1 mL pectin solution (1 wt.%) was added to a 10 mL vial and stirred at 600 rpm. A 1 mL chitosan solution (1.5 wt.%) was added drop by drop at a rate of one drop every 10 sec using a pipette located 10 cm above the surface of the solution with a nozzle diameter of 1.125 μm. The stirring speed increased to 800 rpm as the volume gradually increased and stirred for 40 min. The suspension was treated twice with ultrasound (Ultrasonic Cleaner Manufacturer Expert, Beijing, China) at 70 kHz and 30 °C for 5 min to prevent aggregation. The NPs were stirred at 800 rpm for 1 min between ultrasonic treatments. The prepared solutions were stirred at 600 rpm for 18 h to complete the gelation process.

To prepare NPs labeled with FITC through chitosan, 1 mL of a 2% solution of FITC in methanol and 78 mg of HEPES in 3 mL of deionized water in the dark at room temperature were mixed to a concentration of 0.1 M and stirred at 200 rpm for 1 h, followed by the addition of 15.6 mg EDC and 37.2 mg NHS. This ratio was chosen due to the large amount of amine and amide groups in chitosan; the carboxyl groups of pectin do not need to be activated by EDC in large amounts. The resulting mixture was stirred for 1 h in the dark at 200 rpm. The pH of the resulting solution was measured (should be in the range of 6.7 to 6.9). Next, 0.1 mL of 1 wt.% pectin with a degree of esterification of 10% was diluted in 0.9 mL of the mixture and stirred for 1 h at 200 rpm at room temperature in the dark. The resulting pectin solution (1 mL) and 1 mL of a 2 mg/mL FITC solution (dissolved in DMSO) were mixed by stirring for 12 h at 200 rpm in the dark at room temperature. An orange mixture was formed as a result. Dialysis was performed using a 8–14 kDa membrane for 24 h in 3 mL of distilled water at 4 °C in the dark, with solvent change every 24 h. The dialysis resulted in the removal of FITC not bound to chitosan.

To prepare NPs labeled with FITC through pectin, 78 mg of HEPES in 3 mL of deionized water was dissolved in the dark at room temperature to a concentration of 0.1 M and stirred at 200 rpm for 1 h. Then, 15.6 mg EDC and 37.2 mg NHS were added at room temperature in the dark. This ratio was chosen because of the large number of amino groups in chitosan. Consequently, the carboxyl groups of pectin do not need to be activated by EDC in large amounts. The resulting mixture was stirred for 1 h in the dark at 200 rpm. The pH of the resulting solution was measured (should be in the range of 6.7 to 6.9). Next, 0.1 mL of 1% pectin was diluted in 0.9 mL of the mixture and stirred for 1 h at 200 rpm at room temperature in the dark. The resulting pectin solution (1 mL) and 1 mL of a 2 mg/mL FITC solution (dissolved in DMSO) were mixed and stirred for 12 h at 200 rpm in the dark at room temperature. The same orange-colored mixture was formed as in the process of FITC crosslinking to chitosan. Dialysis was performed using membranes of the same characteristics, for the same period of time, and under the same conditions as in the case of cationic polysaccharide.

The dialyzed solution was centrifuged at 4 °C and 40,000× *g* in the dark for 1 h. The 2 mL suspension was pre-diluted with deionized water to 7 mL. After centrifugation, 2 mL of suspension was left and dispersed 10–15 times using a laboratory pipette in the dark at room temperature. The NPs were filtered with a 10 mL syringe (20 mm cellulose acetate filter with 220 nm pores) under laminar flow to maintain constant NP size and eliminate microorganisms.

### 2.3. Surface Modification by Antibodies

NHS/EDC cross-linking chemistry was used to prepare anti-NCAM1 and anti-Pdl1 antibody-conjugated NPs. The anti-NCAM1 and anti-Pdl1 antibodies were activated by adding NHS and EDC, and then amino-functionalized polyelectrolyte-based NPs were added to the mixture for overnight incubation at 4 °C. Then, the resulting anti-NCAM1 (DF7832, rabbit, titer 1:600) and anti-Pdl1 (PAE788Hu01, rabbit, titer 1:300) antibodies conjugated to pectin–chitosan NPs were centrifuged and washed with PBS several times.

### 2.4. Evaluation of Nanoparticle Fluorescence and Antibody Attachment

The efficiency of NP conjugation with FITC and antibodies was evaluated using a comprehensive experimental protocol. This study utilized the Agilent BioTek Cytation 5 Multi-Mode Cell Visualization and Analysis Reader (Thermo Fisher Scientific, Santa Clara, CA, USA) and Atomic Force Microscopy (AFM) (Bruker, Billerica, MA, USA) for characterization.

The conjugation process with FITC was analyzed at excitation and emission wavelengths of 495 nm and 519 nm, respectively, 1 h after dispensing 20 μL of NP suspensions into a 96-well plate.

Specific antibodies were visualized using secondary antibody labeling with Alexa Fluor 568 goat anti-rabbit IgG (H+L), catalog A11036 (titer 1:2000, recommended by the manufacturer).

The detailed experimental procedure included the following steps:

1. Dispensing 20 μL of NP suspensions onto the bottom of a 96-well plate.
2. Removal of unbound particles.
3. Incubation with a 2 wt.% paraformaldehyde solution in PBS for 15 min.
4. Washing three times with PBS containing Tween-20 (0.1 wt.%) for 5 min each.
5. Addition of 20 μL of PBS containing Triton X-100 (0.05 wt.%) and incubation for 10 min.
6. Subsequent washing three times with PBS-Tween for 5 min each.
7. Incubation with secondary antibodies (anti-rabbit Alexa Fluor 568, A11036, Affinity, China)) diluted 1:2000 in PBS.
8. Final washing three times with PBS-Tween for 5 min each.

Fluorescence intensity was measured using a plate reader at specific wavelength pairs: 495/519 nm for FITC and 579/603 nm for Alexa Fluor 568. All experiments were conducted at 37 °C under dark conditions to ensure optimal preservation of fluorescence signals.

### 2.5. Atomic Force Microscopy

The AFM assay was conducted utilizing a Resolve AFM system manufactured by Bruker (Bruker, Billerica, MA, USA). The analytical procedure encompassed two principal stages: investigation of surface morphology and examination of nanomechanical properties.

Samples were subjected to scanning in PeakForce Quantitative Nanomechanical Mapping (PFQNM) mode for morphological analysis. Measurements were performed with an MSNL-A console (Bruker Optics GmbH & Co., Ettlingen, Germany) in DMEM nutrient medium (Capricorn, Düsseldorf, Germany).

Investigation of nanomechanical characteristics was carried out in PeakForce QNM tapping mode under conditions identical to those employed for morphological analysis. Prior to commencement of the primary analysis, a comprehensive calibration procedure was implemented. This procedure entailed determination of cantilever deflection sensitivity, measurement of the elastic constant via the thermal noise method, acquisition of force curves on a sapphire reference sample, and estimation of the probe radius through scanning of a rough titanium sample. Calculation of probe parameters was executed using Bruker Nanoscope 9.2 analytical software, with the Poisson’s ratio standardized at 0.3 for all measurements.

Standardization of scanning conditions was implemented as follows: scan area of 2 × 2 µm, scanning speed of 0.5 Hz, probe oscillation amplitude of 100 nm, and peak force setting of 500 pN. Analysis of all samples was conducted utilizing Bruker Nanoscope analysis software (version 1.40). All samples were analyzed in liquid medium. The acquired data was utilized for calculation of adhesion and elastic modulus (Young’s modulus) values. Images obtained for NP diameter analysis were processed using ImageJ 1.53t software to quantify NP size.

Prior to AFM investigation, the solution containing NPs was incubated on HCl-treated coverslips for 10 min at room temperature, followed by careful rinsing with sterile deionized water three times. The AFM study was then carried out in a PBS solution, with the AFM probe also immersed in the liquid.

### 2.6. Zeta-Potential Study

Zeta-potential was estimated by photon correlation spectroscopy on a Zetasizer Nano ZS analyzer (Malvern Instruments, Worcestershire, UK). Sample preparation included processing the samples in a Sonopuls HD 3200 homogenizer (Bandelin, Berlin, Germany) at a frequency of 20 kHz. The ultrasound processing time was 3 min. Suspensions with a volume of 1 mL were placed in a DTS0012 cuvette and installed in the thermostable cuvette compartment of the analyzer after ultrasound processing.

### 2.7. Fourier Transmission Infrared Spectroscopy (FTIR)

The studies were carried out on a Shimadzu IRAffinity-1S infrared spectroscope (Kyoto, Japan) with a PIKE Technologies MIRacle 10 (Madison, WI, USA) attachment for attenuated total reflection spectroscopy. The samples were fixed on the plate using a flat pressure tip. The spectra were recorded in the range of 400 to 4000 cm^−1^ with a resolution of 4 cm^−1^ in the absorption mode, with Happ–Genzel apodization.

### 2.8. Raman Spectroscopy

The measurements were performed on an RL1064 device (PHOTON-BIO, Chernogolovka, Russia). The samples were fixed on the plate in 1.5 mL microvessels. The spectra were obtained in the range from minus 5000 to 5000 μm with a resolution of 5 μm in absorption mode, with apodization according to Happ–Genzel. The laser power was 1087 W. The region of 200–4000 μm was analyzed further.

### 2.9. Immobilization of Temozolomide

TMZ was dissolved in 0.4% DMSO to 12 mM for sorption studies. The resulting solution was stirred for 1 day at 25 °C until the chemotherapeutic drug was completely dissolved. A completely dissolved drug form was taken as one in which no precipitation was observed when centrifuged at 15,000× *g* for 15 min.

The solution was added to the pectin and chitosan NP suspension (final TMZ concentration of 4 mM) after ultrasound treatment in the dark at room temperature with stirring at 600 rpm for 90 min (reaching ultimate adsorption and reaching plateau).

The efficiency of immobilization and release of molecules was investigated using a UV-1900 ultraviolet-visible (UV) spectrophotometer (Shimadzu Co., Kyoto, Japan). The concentration of TMZ was determined by measurement at a wavelength of λ = 330 nm [18]. The optical density was measured at intervals after the addition of the chemotherapeutic agent to the NPs. The samples were taken into tubes and centrifuged at 40,000× *g* for 5 min, after which 10 µL of supernatant was taken each time. The optical path length was 1 mm, and 10 μL of solvent (0.4% DMSO) was used as a standard.

The experimental data was analyzed against three different mathematical models and some kinetic parameters of TMZ loading were obtained.

### 2.10. Evaluation of Nanoparticle Transport Through a Model Blood Vessel

A section of the bloodstream of the lower extremities obtained from patients at the Medical Center of the Far Eastern Federal University was used as a model of the BBB. There were no exclusion criteria for patients. Lower limb vein samples up to 10 cm in length and 1.2 mm in lumen width were taken from each patient on admission. This study was approved by the Local Ethics Committee of Far Eastern Federal University (Protocol No. 13 dated 26 November 2024) and was conducted in accordance with the principles of the Helsinki Accords. Each participant signed a written informed consent form. The vessel was cut into sections up to 7 mm in length, opened from the inside with the endothelial layer upward, and placed on a section of Petri dish (35 mm) coated with cyanoacrylate glue. These operations were performed under laminar flow. The vessel sections were incubated for 30 min in 5 mL of culture medium.

As part of the study of NPs and blood vessel model interaction, NP accumulation in the vessel was assessed using laser scanning confocal microscopy (LSM) (Olympus Fluoview, Center Valley, PA, USA), and NPs were localized in the vessel cells using LSM and scanning electron microscopy (SEM). The presence of NPs in the vessel was confirmed using Raman spectroscopy.

Accumulation of NPs in the vessel was evaluated by LSM microscopy. 3D images were captured by LSM using a straight module and ×25 objective lens at excitation/emission wavelengths of 495/519 nm for FITC. After vessel imaging, the medium was replaced with one containing 10% by volume of the NP suspension and incubated at 37 °C and 5% CO_2_ for an 1 h. Imaging was repeated with the same parameters. The samples were fixed with 4% paraformaldehyde solution for 30 min, washed three times with PBS-T for 5 min each, and encapsulated in PBS-Azide (0.4%). The samples were washed three times with PBS-T for 5 min each and encapsulated in PBS. Measurements were carried out on an RL1064 device.

The position of NPs in the vessel was evaluated by LSM with Rhodamine 6G and Hoechst 33342 staining and confirmed by high-resolution electron microscopy using an Ultra Plus scanning electron microscope (Carl Zeiss, Oberkochen, Germany). For LSM imaging, the sample was washed with PBS, 3 mL of Rhodamine-6G solution with a concentration of 6 × 10^−6^ M in DMEM was added and incubated at 37 °C and 5% CO_2_ for 2 h in the dark, and then Hoechst 33342 at a dilution of 1:1000 was added and incubated for 15 min in the dark. 3D images were captured by LSM using a straight module and ×25 objective at excitation/emission wavelengths of 495/519 nm for FITC, 535/565 nm for Rhodamine-6G, and 355/461 nm for Hoechst 33342. After vessel imaging, the medium was replaced with one containing 10% by volume of the NP suspension and incubated at 37 °C and 5% CO_2_ for an 1 h. Imaging was repeated with the same laser and detector parameters.

### 2.11. Scanning Electron Microscopy

Before LSM imaging using SEM, the bloodstream cross-section samples were lyophilized and dried. After lyophilization drying at minus 100 °C and 0.008 mBar, the outer surface of the samples was coated with a nanometer layer of gold. SEM images were obtained using a field emission SEM Ultra Plus (Carl Zeiss, Oberkochen, Germany). Observations were carried out at the accelerating voltage of 10 kV and vacuum of 10^−5^ mm of mercury. An InLens detector was used during the investigations.

### 2.12. Analysis of Nanoparticle Cytotoxicity Toward Brain Cancer Cells

Human malignant glioblastoma U87-MG cells were maintained in Dulbecco’s modified Eagle medium (DMEM) supplemented with 20 mM L-glutamine, 4.5 g/L glucose (Paneco, Moscow, Russia), 10% (*v*/*v*) fetal bovine serum (Cytiva, Logan, UT, USA), 50 μg/mL penicillin, and 50 μg/mL streptomycin (Paneco, Moscow, Russia) at 37 °C in 5% CO_2_ atmosphere. The medium was changed every 72 h. Cells were harvested after reaching 70% confluency by trypsin, followed by washing with 1×PBS buffer, resuspending, and using for experiments.

The human malignant glioblastoma U87-MG cell line was obtained from American Type Culture Collection (Manassas, VA, USA). The cell line was tested regularly to confirm the lack of mycoplasma infection with the mycoplasma detection kit MycoReport (Evrogen, Moscow, Russia).

U87-MG cells were plated in 24-well plates (12,000 per cm^2^) and incubated overnight under standard conditions for attachment. The nutrient medium was then replaced with fresh medium, to which the NP suspension was added (10% of the total volume). The control group was given only the solvent (0.4% aqueous DMSO solution acidified with acetic acid to pH 7.4) instead of NPs.

The effect of NPs, including those loaded with TMZ, on the proliferation of U87-MG cells was assessed using a Cell-IQ automated cell culture and analysis system (CM Technologies Oy, Jyväskylä, Finland). The cells were visualized in nine fields of view for each sample by phase contrast using a Nikon Plan Fluor 10× objective (Yokohama, Japan). Visualization of each field of view was carried out with an interval of 2 h.

The Cell IQ Cell Finder algorithms for machine vision-based cell shape recognition were tuned by automatically calculating an average cell image from a manually generated library. Three sample cell libraries (triangular, spindle, and round) and two separate libraries for dead cells and cell debris were created.

### 2.13. Statistical Analysis

Statistical analysis of the data was performed using Student’s *t*-test. Differences were considered significant at a *p* value < 0.05 and most significant at a *p* value < 0.01. Statistical analysis was performed using GraphPad Prism ver. 10 software (GraphPad Software, San Diego, CA, USA). Statistical differences were considered significant if *p*-values were less than 0.05 (* *p* ≤ 0.05) and highly significant if *p*-values were less than 0.01 (** *p* ≤ 0.01), less than 0.001 (*** *p* ≤ 0.001), or less than 0.0001 (**** *p* ≤ 0.0001). The Dunnett T2 test was used to compare values with the control group. Nonparametric analysis methods were used to quantify differences between the groups: Dunnett T3, Dunn, and Kruskal–Wallis multiple comparison tests and the two-step linear enhancement procedure of Benjamini, Krieger, and Yekutieli. One-way analysis of variance (ANOVA) was used to compare three or more groups.

## 3. Results

### 3.1. Nanoparticle Synthesis and Characterization

Polyelectrolyte complexes (PECs) of cationic chitosan and anionic pectin were prepared by the ion gelation method. The stoichiometric ratios of the two polysaccharides belonging to the aggregation type and corresponding to the most stable and reproducible suspensions based on our previous work were used [7]. A complete list of obtained NPs with their modifications is shown in Table 1.

**Figure 1:**
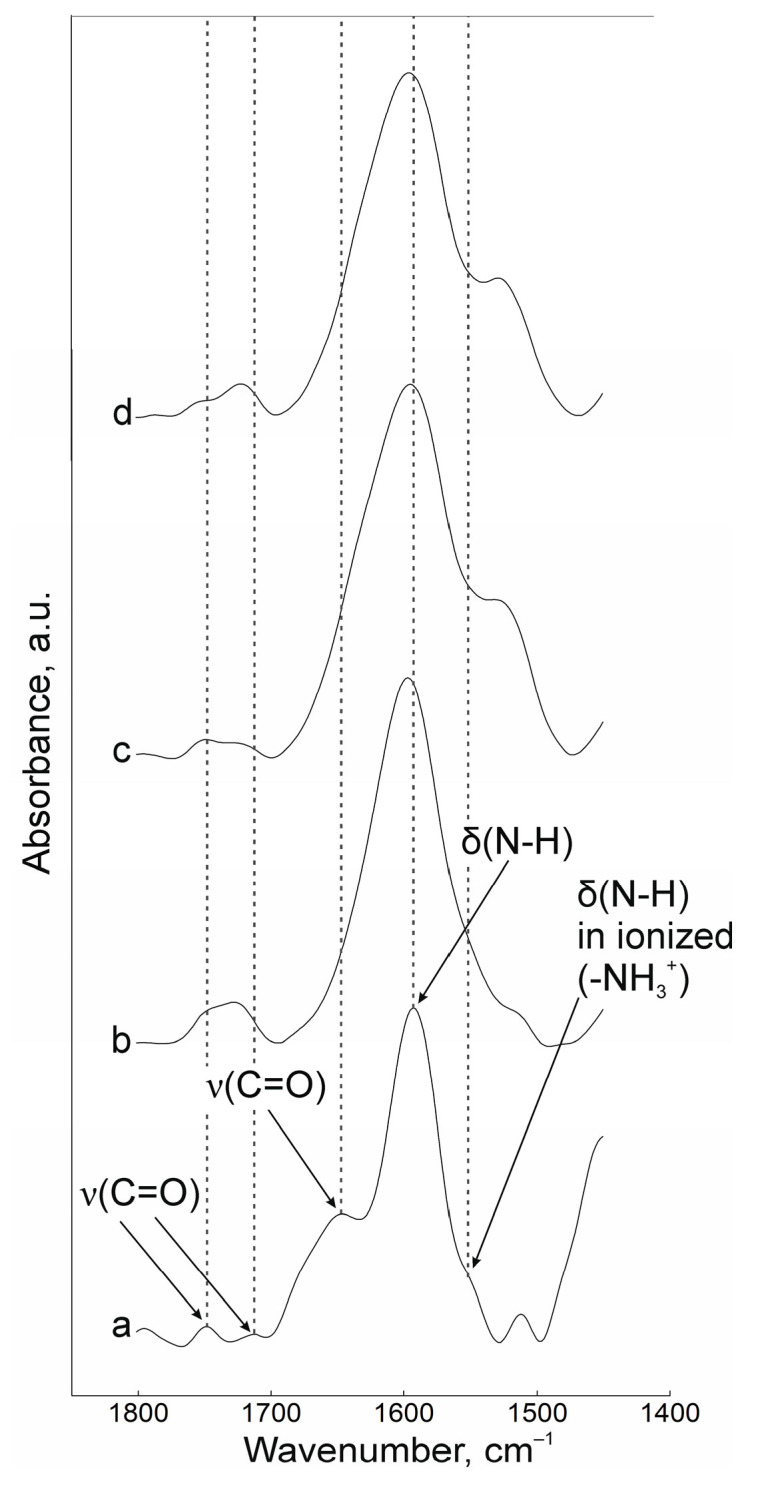
FTIR spectra of powders of (a) chitosan and (b) pectin, (c) NPs-01-01*, (d) NPs-01-05.

Interactions between oppositely charged macromolecules are based on predominantly electrostatic interactions. The FTIR spectroscopy data indicates that cross-linking during gelation is carried out by amino groups of chitosan and carboxyl groups of pectin. The results of measurements in the range of 1450 to 1800 cm^−1^ are presented in Figure 1. In the spectrum of the initial chitosan powder (Figure 1a), the characteristic peaks of the valence vibrations of the ν(C=O) groups are located at 1750, 1714, and 1650 cm^−1^, and the peak of the deformation vibrations of δ(N-H) is at 1592 cm^−1^. The band at 1555 cm^−1^ belongs to the protonated form of the amino group. The above band shifts to 1525 cm^−1^ upon the interaction of chitosan and pectin during fabrication of the nanoscale complexes (Figure 1c,d) and shifts further into the low-frequency region the more cationic polyelectrolyte is contained in the initial composition. In the spectra of NP suspensions, the positions of the C=O bond peaks also shift slightly with decreasing values of the wavenumber. The ν(C=O) band of the initial pectin (Figure 1b) shifts from 1730 to 1715 cm^−1^. Correlation of the lines was carried out on the basis of [36,37,38].

**Figure 2:**
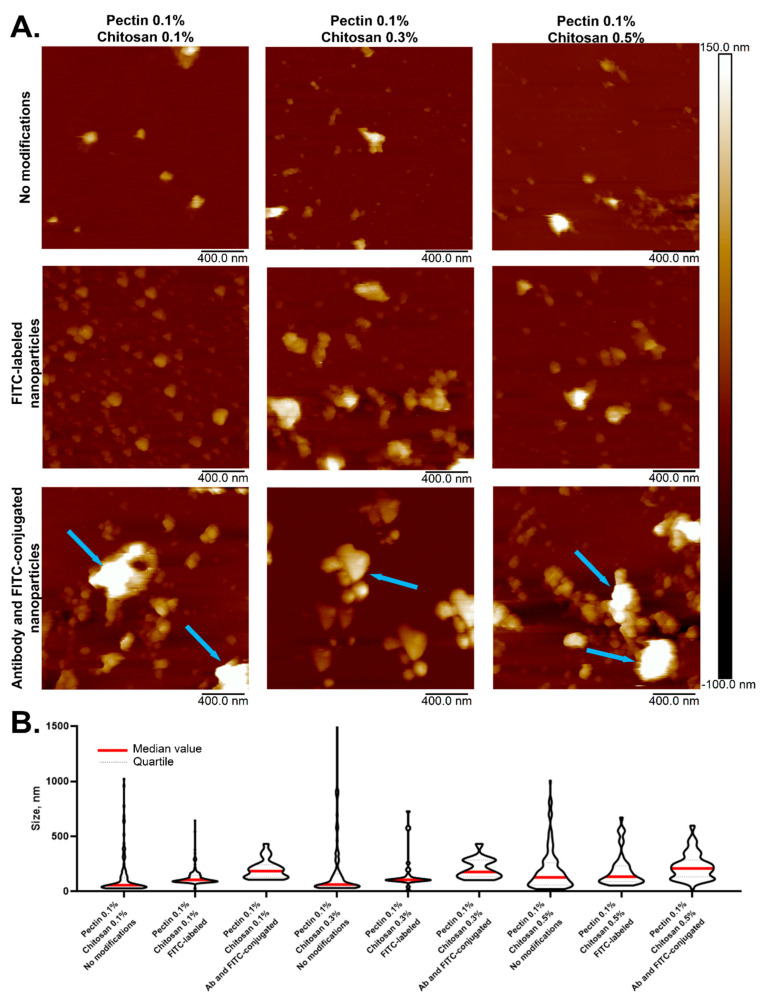
NPs with various polysaccharide compositions, modified with FITC and antibodies. (**A**) NP representative images. Blue arrows show the NP conglomerates; (**B**) NP size comparison.

The shape, size, and zeta-potential of the particles were investigated during their preparation and surface modification. These parameters should primarily influence the targeting and cellular uptake of NPs with the drug [39]. Samples with different stoichiometric ratios of chitosan and a constant concentration of 0.1 wt.% pectin were obtained. It is this concentration of pectin that allows obtaining individual colloidal particles. Chitosan as a source of counterions changes the size of the particles, keeping them stable for at least 3 days from the time of production. AFM images are shown in Figure 2. The initial particles are represented by ratios of pectin to chitosan of 0.1 wt.%:0.1 wt.% (NPs-01-01), 0.1 wt.%:0.3 wt.% (NPs-01-03), and 0.1 wt.%:0.5 wt.% (NPs-01-05).

The NPs were modified by attaching specific antibodies to increase permeability across biological membranes. These antibodies target two proteins, PDL-1 and NCAM, which are known to be overexpressed in GBM. Additionally, to monitor the NPs both in living organisms (in vivo) and under laboratory conditions (in vitro), we labeled them with FITC [40]. The initial NPs exhibited a median diameter range of 56 to 209 nm. A notable trend was observed: the diameter increased as the chitosan concentration in the starting mixture rose.

Unmodified NPs exhibited the smallest diameter compared to their modified counterparts (Figure 2). While increasing the chitosan concentration did not significantly enlarge NP size, it did induce a tendency towards the formation of NPs conglomerates. Modification with both FITC and antibodies further exacerbated this conglomerate formation, as illustrated by the largest aggregates marked with blue arrows (Figure 2A). A clear size increase trend was observed during NP modification: baseline unmodified particles were the smallest, FITC labeling caused an enlargement that was particularly noticeable at 0.1% and 0.3% chitosan concentrations, and antibody attachment resulted in a significant size increase, irrespective of polysaccharide concentration levels (Table A1 in Appendix A).

A direct correlation was found between the concentration of cationic polyelectrolyte in the initial composition and the average diameter, with increases ranging from 15% to 35%.

Zeta-potential evaluation of the colloidal systems showed an increase in the values of this parameter along with an increase in the chitosan concentration of the samples. This may correspond to an increase in the number of free dissociated amino groups in PEC. In the case of the original particles, the particles with FITC, the particles with antibodies, and complexes with fluorescein and protein, the zeta-potential varied in the range from 24.4 to 31.2, from 25.1 to 36.7, from 12.6 to 27.9, and from minus 9.2 to 2.4, respectively. Zeta-potential decreased for all samples with antibody injection, regardless of whether native or fluorescein-labeled pectin was used. In general, most of the samples showed a positive surface charge, which was indicated at the beginning of the experiment as an advantage for potential drug delivery systems.

**Figure 3:**
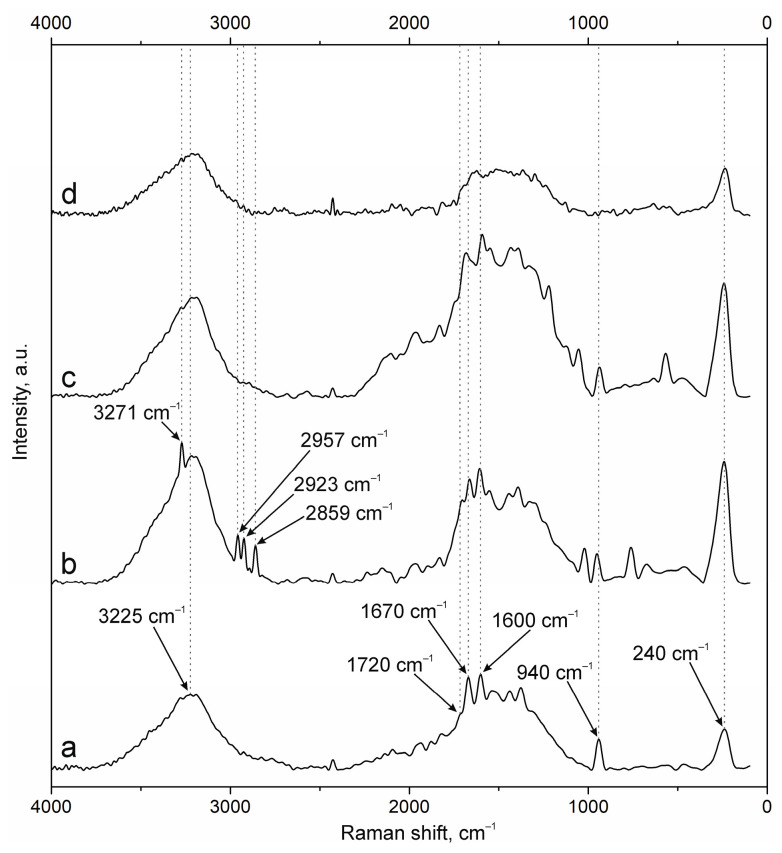
Raman spectra of (a) NPs-01-01, (b) NPs-01-01-FITC, (c) NPs-01-01-Ab, and (d) NPs-01-01-FITC-Ab.

The results of NP surface modification were analyzed by Raman spectroscopy. The positions of the characteristic bands of the components of PEC [38,41,42,43] and additional substances, including the fluorescent label FITC and groups of antibodies to NCAM-1 and PDL-1 receptors, were determined. A group of bands in the spectrum of suspension NPs-01-01 (Figure 3a) at ~3225 cm^−1^ refers to vibrations of hydroxyl groups in various positions and hydrogen bonds, valence vibrations ν(N-H) in NH_3_ at 3191 cm^−1^, and ν(C=O) in carboxyl groups and acetylated monosaccharide residues at 1720 and 1670 cm^−1^, respectively. The intense band around 1600 cm^−1^ includes the δ(N-H) bond vibrations of amino groups and (C=O) carboxyls in protonated form. The δ(N-H) line of dissociated amino groups is also displayed. The ν(C-O-C) fluctuation in glycosidic bonds is detected at 941 cm^−1^. The δ(C-NH-C) band of acetylated macromolecular residues is well expressed in the low-frequency region. The position and correlation of other bands are given in Table A2.

Embedding of FITC (Figure 3b) leads to the appearance of additional bands in the spectrum. The main lines are located at 3271 cm^−1^ (ν(O-H)), 2957, 2923, and 2859 cm^−1^ (ν(C-H)), and 1503 cm^−1^ (skeletal in-plane vibrations of aromatic ring). The peak at 1296 cm^−1^ is presumably attributed to phenoxide ion stretching conjugated with xanthene ring stretching in FITC. Surface cross-linking of antibodies (Figure 3c) leads to a significant increase in the peak group in the range of 1000 to 2000 cm^−1^. This is due to strong interactions through carboxyl groups and the participation of amino groups in cross-linking. The cross-linking of protein structures to NPs already containing FITC (Figure 3d) reduces the intensity of all bands characterizing fluorescein. This may have a further effect on the decrease in fluorescence intensity of NPs of this composition.

**Figure 4:**
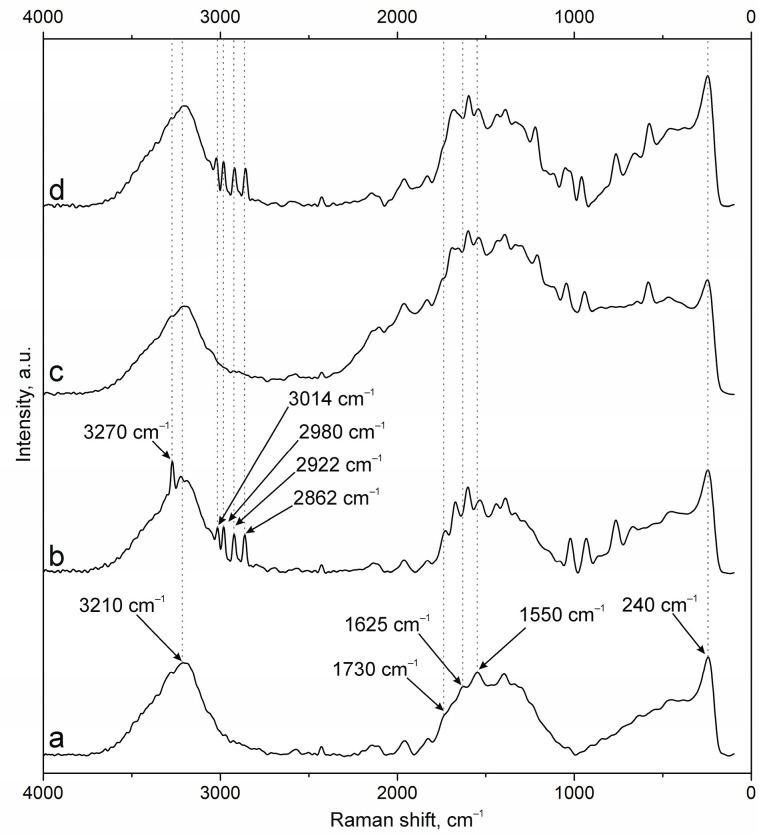
Raman spectra of (a) NPs-01-03, (b) NPs-01-03-FITC, (c) NPs-01-03-Ab, and (d) NPs-01-03-FITC-Ab.

**Figure 5:**
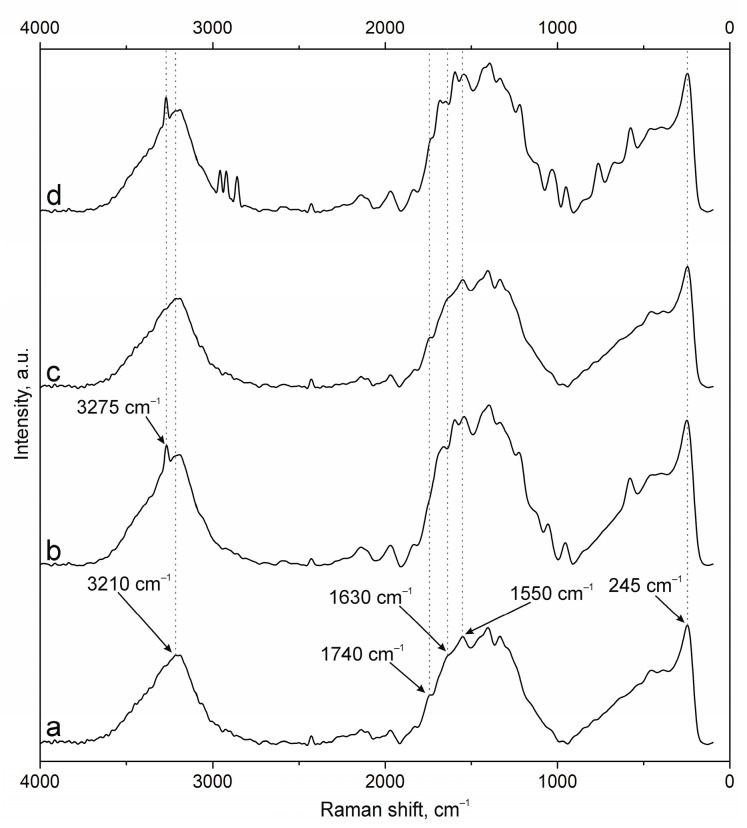
Raman spectra of (a) NPs-01-05, (b) NPs-01-05-FITC, (c) NPs-01-05-Ab, and (d) NPs-01-05-FITC-Ab.

The spectra of the original samples NPs-01-03 (Figure 4a) and NPs-01-05 (Figure 5a) also contain the above-mentioned peaks characteristic of the original chitosan and pectin. Their position does not change in the spectra of the modified particles (Figure 4d and Figure 5d). FITC cross-linking to NPs-01-03 (Figure 4b) is accompanied by the appearance of the narrow and intense bands for ν(O-H) at 3290 cm^−1^, ν(N-H) at 3014 cm^−1^, and ν(C-H) at 2980, 2922, and 2862 cm^−1^. The marked vibrations are conserved in position for NPs-01-03-FITC-Ab. In the case of sample NPs-01-05-FITC (Figure 5b), only the ν(O-H) peak at 3275 cm^−1^ is visible, but the spectrum of NP-01-05-FITC-Ab also shows ν(N-H) and ν(C-H) bands. Perhaps this will further explain the large fluorescence intensity of these samples. The bands in the spectra of all complexes based on NPs-01-03 (Figure 4b–d) and NPs-01-05 (Figure 5b–d) strongly increase the intensity of the peak group in the range of 1000 to 2000 cm^−1^. This may also indicate efficient and strong cross-linking of polysaccharides with fluorescein and antibodies. All changes in the position of the main bands can also be observed in Table A3 and Table A4 corresponding to NPs-01-03- and NPs-01-05-based complexes, respectively.

### 3.2. Temozolomide Loading Results and Nanoparticle Cytotoxicity

The system typically demonstrates a rapid initial absorption phase, followed by a gradual slowdown in loading rate. This deceleration is primarily due to the increasing distance the drug molecules must travel to diffuse out of the external medium. Thus, the bulk of the drug is absorbed within 5 min and does not depend on the ratio of polysaccharides in the particle. Then the process slows down, and in 15 min the absorption curve practically reaches a plateau.

**Figure 6:**
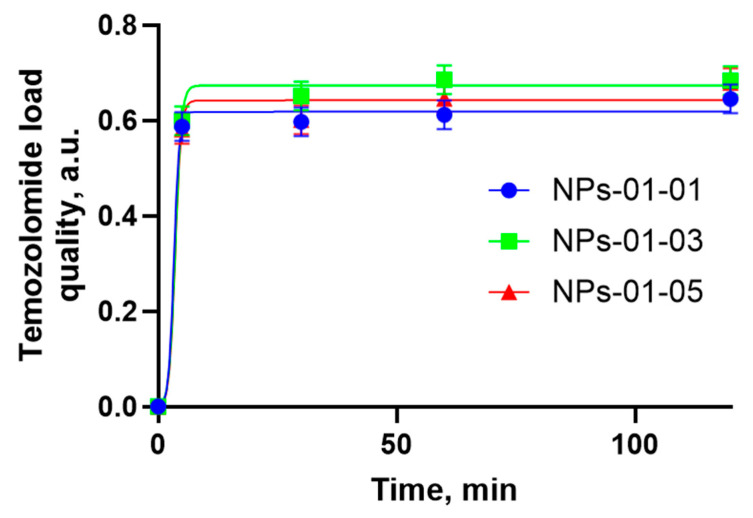
TMZ concentration profiles in the pectin–chitosan NPs as a function of time for various ratios of polysaccharides: NPs-01-01 (blue circle), NPs-01-03 (green square), and NPs-01-05 (red triangle).

A slightly greater sorption of TMZ by NPs with a ratio of 1:3 is observed. This may be due to the mutual repulsion of the like-charged groups included in the gel network. Different combinations of pectin and chitosan results in changing sizes and structures of the gel. Perhaps the ratio 1:3 is the best combination for TMZ immobilization in experimental pectin–chitosan particles. The characteristics of TMZ sorption from medium into chitosan–pectin NPs are shown in Figure 6.

**Figure 7:**
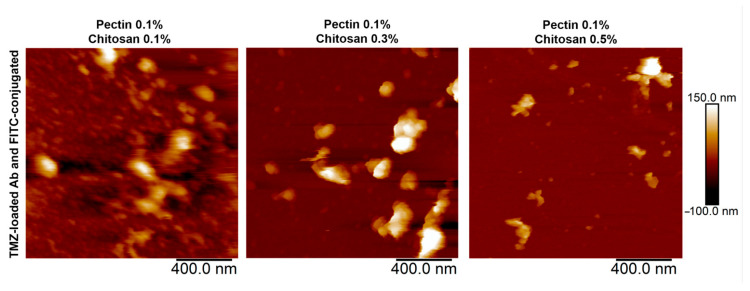
TMZ-loaded polyelectrolyte NPs functionalized with FITC, NCAM, and PDL-1 antibodies.

After loading with TMZ, the produced NPs did not show significant changes in their morphology compared to the unloaded NPs. Additionally, the TMZ-loaded NPs retained their conglomerate-like structures (Figure 7).

**Figure 8:**
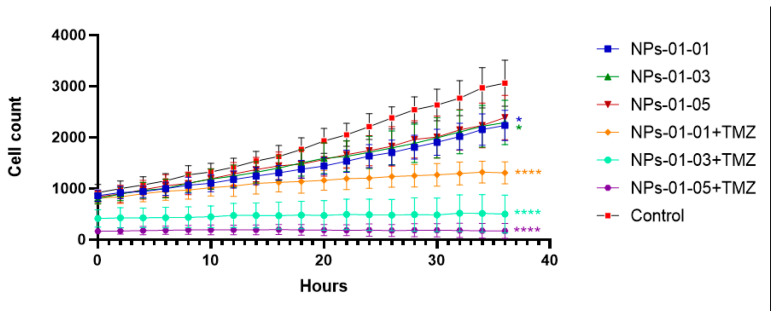
Growth curve of U87-MG cells treated with NPs. Mean ± SD, n = 4. Statistical differences were designated as significant if the *p*-values were less than 0.05 (* *p* ≤ 0.05) or less than 0.0001 (**** *p* ≤ 0.0001). Dunnett’s multiple comparisons test was used to assess differences between each of a number of treatments and a control.

The effects of the NPs on the U87-MG glioblastoma cell line were evaluated using a Cell-iQ automated cell culture and analysis system. As shown in Figure 8, chemotherapeutic agents, as well as NPs with and without TMZ, except NP-01-05, significantly retarded cell proliferation. After 1.5 days from the beginning of the experiment, the proliferation of glioblastoma cells that were injected with empty particles decreased by 1.32 ± 0.11 times compared to the control group. Treatment of cells with TMZ at concentrations of 200 and 400 μM, as well as TMZ-loaded NPs, resulted in the death of more cells within the first 20 min, leaving the number of cells exposed to NP-01-03 and NP-01-05+TMZ unchanged. Treatment of U87-MG cells with NP-01-01-01+TMZ resulted in a 3.82-fold decrease in proliferation compared to the control. As shown earlier, these materials have the lowest efficiency of TMZ sorption, which may explain the resulting biological effect. The morphology of cells treated with unloaded NPs of different composition practically did not change: they remained spindle-shaped and triangular, branches were present, and cell distribution remained uniform. Cells treated with TMZ, including NPs loaded with TMZ, changed their shape to rounded, and there was a sharp decrease in size, destruction of outgrowths, decrease in the size of nuclei, as well as the formation of apoptotic bodies near the cells.

### 3.3. NP Surface Modification

**Figure 9:**
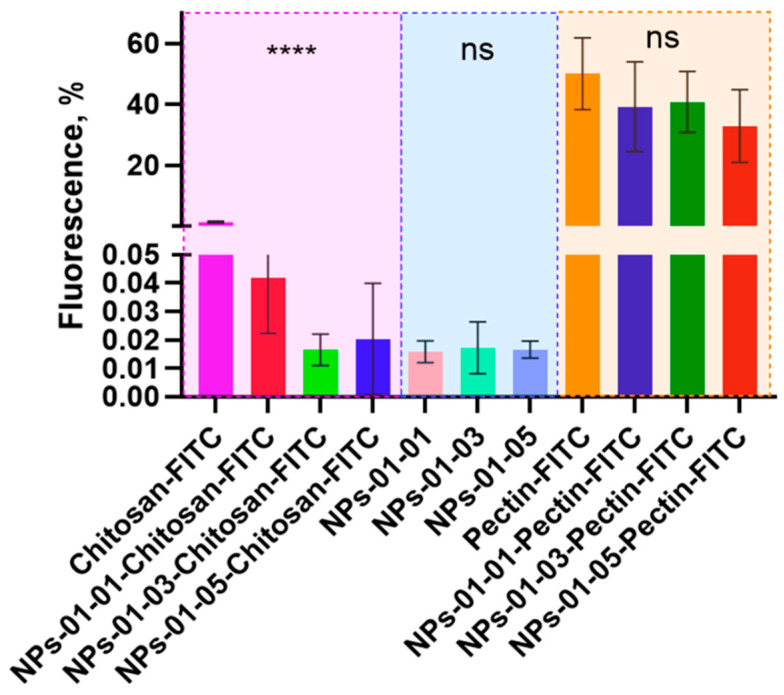
Fluorescence of FITC solutions, FITC-labeled polymers, and FITC NPs. The area with FITC-labeled chitosan and its nanomaterials is highlighted in purple, the area with unlabeled NPs is highlighted in blue, and the area with FITC-labeled pectin and its nanomaterials is highlighted in purple Statistical differences were designated as significant if the *p*-values were less than 0.0001 (**** *p* ≤ 0.0001), *p*-values more than 0.05 (* *p* ≤ 0.05) attributed to no significant (ns). The Kruskal–Wallis test was used to assess differences between two independent samples.

The NPs were labeled with FITC for further visualization in biosystems. The relative fluorescence results obtained using a multi-mode tablet reader were converted to percentages, where the relative fluorescence of the FITC solution (2 wt.%) added to the polymers was taken as 100% (Figure 9). Conjugation was performed both through chitosan, without binding of the polymer to the dye by chemical linkers, and through pectin, with binding by a mixture of EDC and NHS. In the first approach, the fluorescence of the polymer was 1.37%, while in the second approach it was 50%. This may suggest that linkerless crosslinking directly through the amino groups of chitosan is either inefficient or is accompanied by quenching of dye fluorescence. When NPs based on labeled polyelectrolytes are formed with unlabeled ones, the fluorescence drops in all cases: for NPs based on labeled chitosan, it drops by 0.015–0.04%, and by 32.8–40.7% for those based on labeled pectin. The average fluorescence values of NPs based on unlabeled polyelectrolytes were 0.015–0.017%. The differences in fluorescence of the labeled chitosan-based materials were statistically significant (*p* value < 0.0001), in contrast to pectin-based particles, where the differences were not statistically significant (*p* value = 0.0708). In further work we used the technique of conjugation of NPs with fluorophores through pectin.

**Figure 10:**
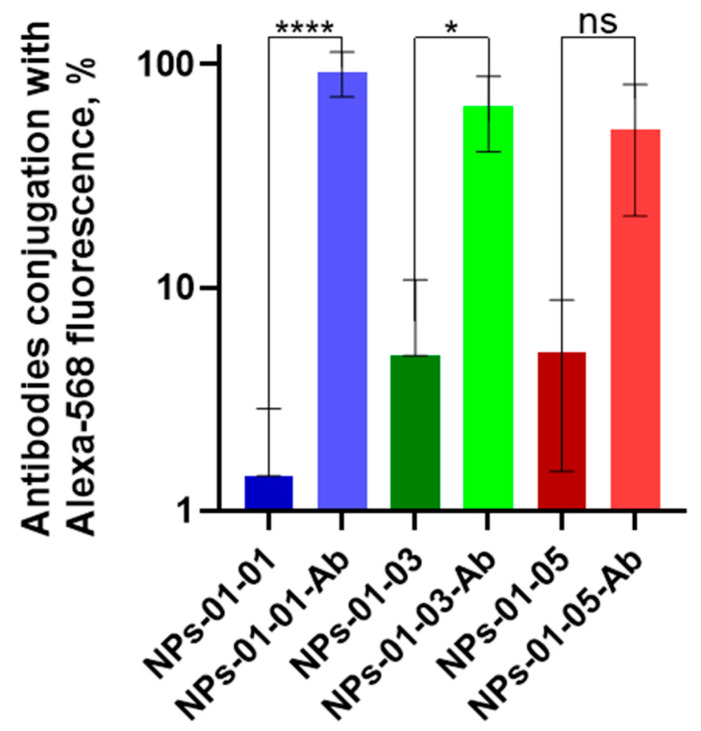
Fluorescence of NPs functionalized with antibodies to NCAM-1 and PDL-1 receptors and labeled with Alexa-568. Both of these proteins are markers of GBM and are not expressed by cells in their microenvironment. Statistical differences were designated as significant if the *p*-values were less than 0.05 (* *p* ≤ 0.05) or less than 0.0001 (**** *p* ≤ 0.0001), *p*-values more than 0.05 (* *p* ≤ 0.05) attributed to no significant (ns). Dunn’s test was used to assess differences between two independent samples.

Antibody crosslinking was performed through a similar method: conjugation via EDC and NHS. Modification was performed for NPs both with and without FITC. Figure 10 shows the change in fluorescence of the primary and secondary antibody conjugate on the NP surface. The fluorescence of the initial solution of the primary antibody–secondary antibody conjugate is taken as 100%. The fluorescence increased from 1.4–5.2% to 51–92%. Thus, all tested particles after conjugation with primary antibodies demonstrated reliable binding to NPs.

### 3.4. Nanoparticle–Endothelium Interaction

An experimental model using human venous tissue obtained by biopsy was designed to study the interaction of NPs with the BBB. Vascular tissue was placed on a culture dish to mimic the BBB system and to assess the penetration and distribution of NPs of different compositions.

**Figure 11:**
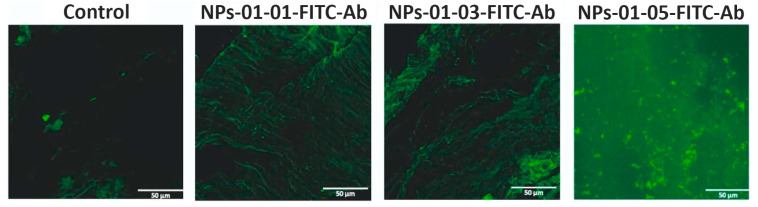
Images of the blood vessel with different samples obtained by LSM. Control—blood vessel without modifications.

Initial fluorescence analysis showed that the native biological material exhibited intrinsic autofluorescence in the “green” spectrum (495–570 nm), of low intensity. Notably, after exposure to the nanoparticle suspension, a significant increase in the fluorescence signal was found both on the surface and at depths of up to 40 μm (Figure 11).

**Figure 12:**
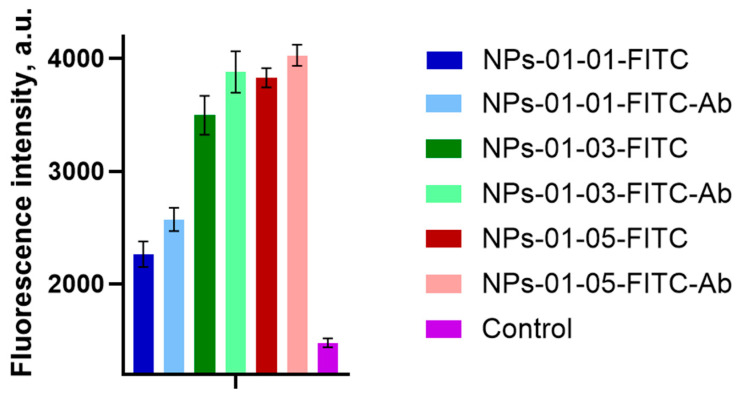
Fluorescence results of NPs on venous wall samples obtained by LSM.

Notably, the fluorescence signal enhancement was most pronounced in the samples treated with NPs with a higher concentration of chitosan, as shown in Figure 12. Statistical evaluation confirmed a highly significant difference (*p* < 0.0001) between the control blood vessel model and those exposed to nanoparticles, as determined by Tamhan’s T2 multiple comparisons test.

Conversely, no statistically significant differences (*p* > 0.9999) were observed between the different NP-treated blood vessel models, as confirmed by Dunnett’s T3 multiple comparisons test. These results are supported by Raman spectroscopy data, which also showed an increase in the peaks corresponding to fluorescein.

This integrated analysis provides valuable information on the penetration and distribution characteristics of NPs in a human vascular model that mimics the BBB environment.

**Figure 13:**
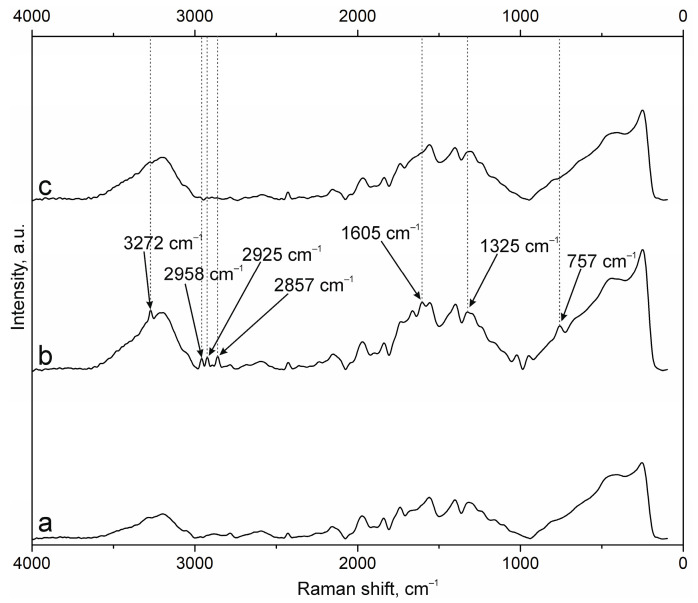
Raman spectra of (a) blood vessel and blood vessel with different samples: (b) NPs-01-01-FITC and (c) NPs-01-01-FITC-Ab.

**Figure 14:**
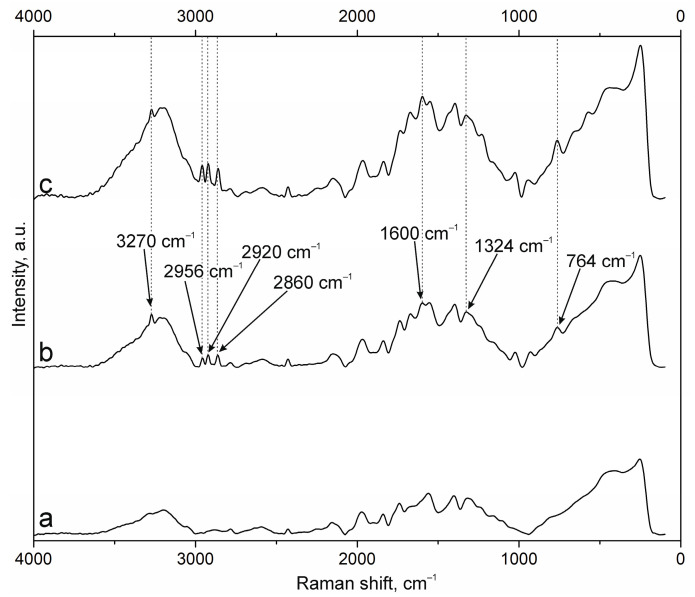
Raman spectra of (a) blood vessel and blood vessel with different samples: (b) NPs-01-03-FITC and (c) NPs-01-03-FITC-Ab.

**Figure 15:**
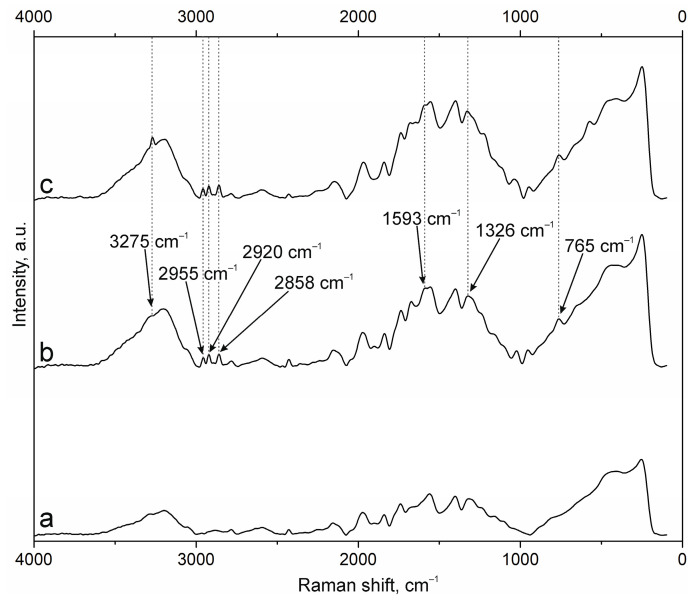
Raman spectra of (a) blood vessel and blood vessel with different samples: (b) NPs-01-05-FITC and (c) NPs-01-05-FITC-Ab.

The above obtained differences in the interaction of particles with the blood vessel are confirmed by Raman spectroscopy. The main bands of FITC vibrations in the case of NPs-01-01-FITC, ν(O-H), ν(N-H), ν(C-H) (three lines), δ(C-C) (two lines), and xanthene ring deformation vibrations, are located at 3272, 2958, 2925, 2857, 1605, 1325, and 757 cm^−1^, respectively (Figure 13). The marked lines are displayed in the case of all types of modified particles in comparison with the spectrum of the empty vessel. The study of complexes containing simultaneously a set of antibodies showed a significant decrease in the intensity of FITC bands in the case of sample NPs-01-01-01-FITC-Ab. This is also confirmed by the weaker fluorescence signal (Figure 12). It can be concluded that it will be difficult to perform tracking of the above samples during in vitro experiments. In the spectra of blood vessel samples treated with NPs-01-03-FITC-Ab suspensions (Figure 14) and NPs-01-03-FITC-Ab (Figure 15), almost all fluorescein bands are conserved in position and intensity in comparison with samples without antibodies.

**Figure 16:**
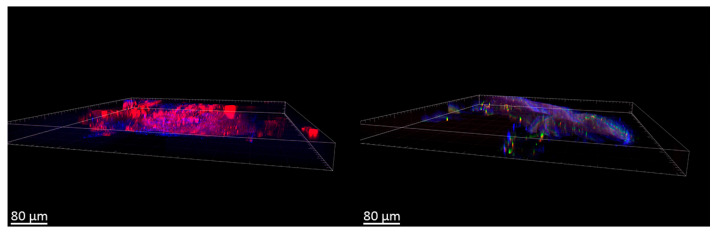
LSM 3D images of the bloodstream before and after treatment with the NPs-01-05-FITC-Ab suspension. Red channel—Rhodamine 6G, blue channel—Hoechst 33342, green channel—FITC.

An attempt was made to visualize NPs in the inner volume of the vessel after the end of the experiment. The blood vessel was stained with Rhodamine 6G and Hoechst 33342. Figure 16 shows the images obtained using LSM. The vessel sample was taken before NPs-01-05-FITC-Ab was added and after. After treatment with NPs, FITC fluorescence was observed, colocalizing with the fluorescence of Rhodamine 6G and Hoechst 33342. Nanomaterials localized both on the surface of the vessel and in the inner layers at a depth of up to 40 μm, mainly forming clusters of NPs.

**Figure 17:**
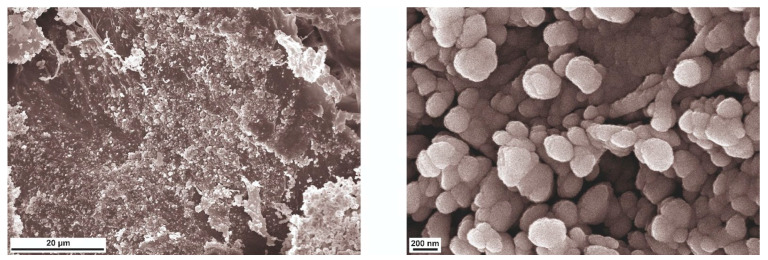
SEM images of the bloodstream cross-section after treatment with the NPs-01-05-FITC-AB suspension. Two magnifications: 1.5KX and 40KX.

SEM imaging showed that the NPs were uniformly distributed over the endothelial layer of the vessel wall. Notably, the nanosized particles showed a strong affinity for the endothelial surface, demonstrating preferential binding to this critical region (Figure 17). Morphometric analysis confirmed that the NPs have a spherical morphology with a mean diameter of 208 nm. These dimensions are in excellent agreement with the size distribution data presented in Table A1, confirming the reproducibility of the material preparation methodology.

## 4. Discussion

NPs for biomedicine should be physiologically compatible (biocompatible), be biodegradable (breakdown in a physiological environment) to physiologically harmless components, or have the ability to be excreted through the kidneys and bile. At the same time, NPs have a number of known advantages. They are too small for sedimentation and are in suspension due to Brownian motion of solvent molecules (water). In addition, they have a large total surface area, and their dispersions provide a high solid content with low viscosity. This allows them to be used as carriers for traditional drugs, proteins, enzymes, vaccines, and antigens. Colloidal delivery systems were originally developed for intravenous administration to enhance their therapeutic activity, overcoming biological barriers and providing controlled drug loading and release, targeted delivery, and prolonged circulation time. Later, other routes of administration were proposed.

Modeling of sorption kinetics by the obtained TMZ-immobilized NPs was carried out. TMZ loading in the initial 2 h was examined using multiple kinetic models. The results showed that the sorption process can be described by first-order kinetic equations (r^2^ = 0.889–0.950) and also closely correlated with the Higuchi model (r^2^ = 0.969–0.997) (Table 2).

First-order is a model that plots a log cumulative amount of drug loaded and time. Higuchi is a model that is plotted as the cumulative amount of drug released and square root of time and assumes that drug loading and release are controlled by diffusion, with absorption being relatively rapid.

**Figure 18:**
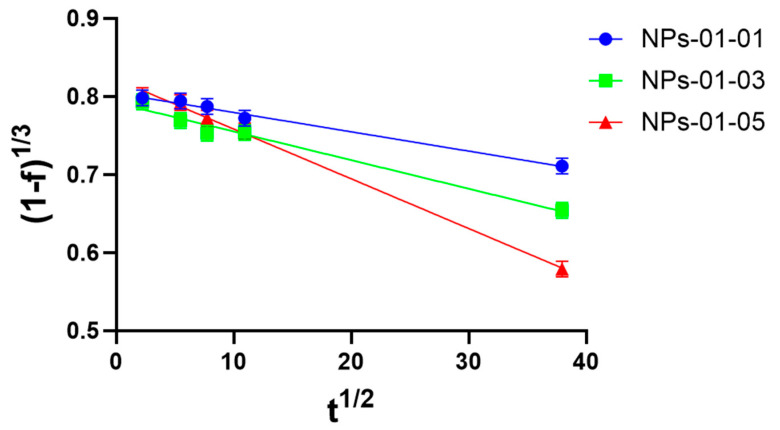
Higuchi plot for pectin–chitosan NPs-01-01 (blue circle), NPs-01-03 (green square), and NPs-01-05 (red triangle). The mean diameters of the NPs were 0.128, 0.169, and 0.196 μm, respectively.

As mentioned in [44], for such systems the kinetic equation can be expressed as a polynomial equation, which is a linear function of the square root of time. A linear plot in coordinates (1 − f)^1/3^ versus t^1/2^ with slope K_r_ is shown (Figure 18).

It can be concluded (Table 3) that the loading process for TMZ can be described by an equation of the form

$${\left(1-f_{t}\right)}^{1/3}\;\text{versus}\;t^{1/2},$$

where f_t_ is the fraction of material loading during time, and K_r_ is the loading rate constant, which is consistent with data from other researchers [45]. In most cases, the intra-particular diffusion is the slowest, rate-limiting step of the sorption process, which can be fitted into the mathematical Nakai–Tachikawa model. The solution of this differential equation is given by the following equation:

$$f\left(\frac{q_{t}}{q_{m}}\right)=\frac{4\cdot \pi^{2}\cdot D_{t}\cdot t}{2.3\cdot d^{2}}$$

where q_t_ is the concentration of the drug in the particles at time t, q_m_ is the concentration of the drug in the particles at equilibrium (t), and d is the mean particle diameter. Plotting $f\left(\frac{q_{t}}{q_{m}}\right)$ versus time allows one to determine the global diffusion coefficient if intra-particle diffusion is the limiting step of the drug loading process. The results of the experiment confirm that the process closely correlates with the Higuchi model (r^2^ = 0.981–0.997) and the Nakai–Tachikawa model (r^2^ = 0.956–0.996) (Table 3).

**Figure A1:**
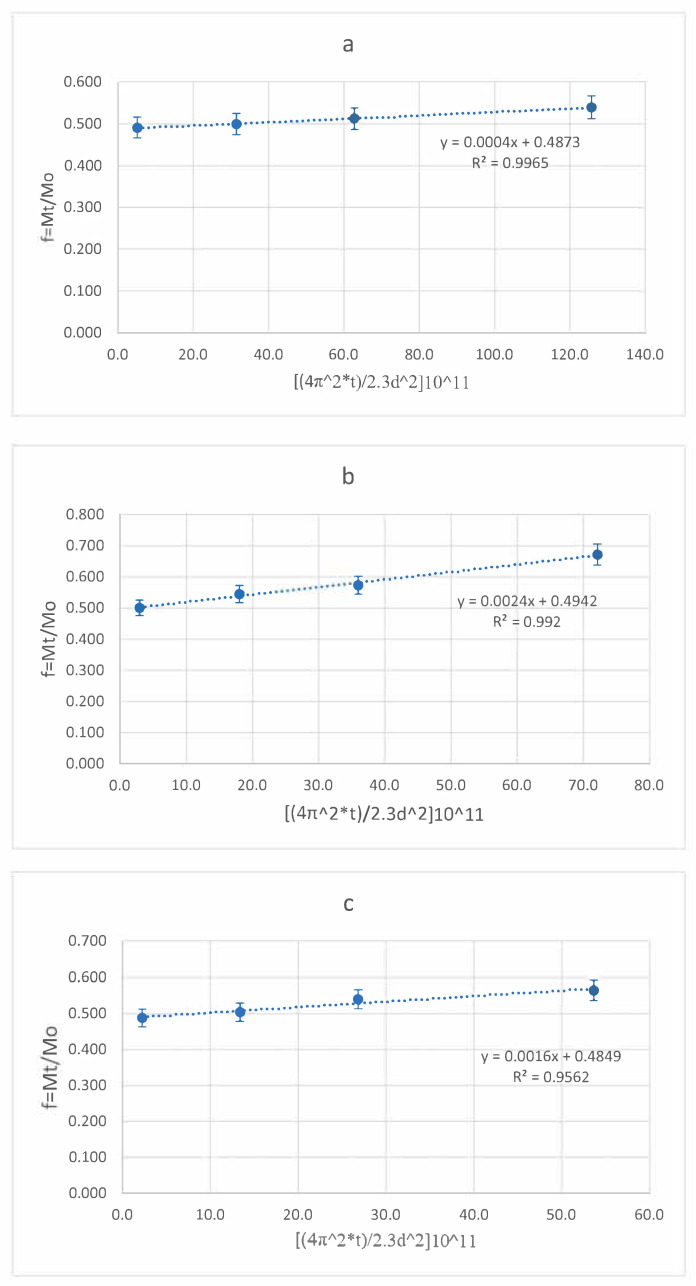
N-T model for NPs: (**a**)—NPs-01-01, (**b**)—NPs-01-03, (**c**)—NPs-01-05.

Therefore, from the linear plot (Figure 18), the loading constant of TMZ loading to pectin–chitosan particles were obtained. The diffusion constant, D, was calculated according to two mathematics models using linear regression (Figure A1). They are shown in Table 4.

Assuming that the process of drug reduction in solution is related to both the process of drug sorption by particles and drug diffusion within the particle, the absorption/diffusion number was calculated (ADN). It expresses the relative importance of sorption and diffusion terms with respect to the overall loading process. The results showed a predominant role of diffusion in sample NPs-01-03 (ADN = 6.62), while the kinetics of the process with NPs-01-01 are mainly associated with primary sorption of the drug from the solution (ADN = 26.9). Therefore, the loading profile of TMZ can be controlled by changes in the conformation of pectin–chitosan NPs and the ratios of polysaccharides in them.

Different effects depending on the physical and chemical properties of the delivery systems, the presence in the model of cells from the BBB and microenvironment, the type of tumor, and the delivered chemoactive drugs were observed in the studies of the effect of pectin and chitosan particles on tumor cells.

NPs potentially promoting invasion of tumor cells by drug delivery through the BBB have been studied for a long time [46,47,48], but PECs based on pectin and chitosan aimed at the treatment of GBM are not often found. Particles with a median diameter range from 56 to 209 nm (average diameter from 128 to 195 nm) and a zeta-potential from 24.4 to 31.2 mV were obtained in our work. The Young’s modulus of the particles was 129–164 kPa, because of which the nanomaterials can be classified as hard enough for cellular entrapment and soft enough for long-term accumulation on the cell surface. The adhesion of the NPs samples ranged from minus 3.2 to minus 0.7 pN, due to which we assume that these structures have a high content of charged functional groups on the surface, due to which a high modulus surface charge (in our case cationic) is formed. This allows particles to accumulate in large quantities at dense contacts between endotheliocytes and to pass the BBB by paracytosis based on the literature data [49].

When FITC NPs were modified, their sizes did not change significantly. At the same time, these colloidal structures showed fluorescence comparable to that of the initial FITC solution, which made them suitable for further bioimaging.

After modification of the NP surface with antibodies, a correlation of approximately a 20% increase in diameter was observed for all NP types. This may be confirmation that the antibodies anchored on the surface of the NPs. Immunofluorescence analysis showed that the fluorescence of the conjugate of NPs modified with primary and secondary antibodies was comparable to the fluorescence of the conjugate of the original primary and secondary antibodies. This was also confirmation that the antibodies were not in the NP suspension, but on the surface of the NPs, because the suspension was pre-centrifuged at 40,000× *g*, at which antibodies do not settle. Another confirmation of conjugation of the NPs with the antibodies was the appearance of peaks corresponding to the antibodies on the Raman spectra of such materials. Also, after conjugation with antibodies, Young’s modulus either did not increase or increased two times, and also adhesion increased to minus 0.28 and minus 0.02, close to zero. This may be due to the increased heterogeneity of the nanomaterial surface after conjugation with antibodies. The zeta-potential of NPs after antibody functionalization decreased for all types of NPs, but the values by which the decrease occurred differed significantly among samples. This can be explained by the heterogeneity of the Ns surface, as well as by the different amounts of antibodies that have adhered to the NP surface.

Sorption of TMZ by the particles also altered the physicochemical properties of the nanomaterials. A significant decrease in size by 10% was observed for NP-01-03, in contrast to other samples, where the size did not significantly increase or did not change. Young’s modulus of chemo-loaded NPs increased most significantly by about 2-fold for NP-01-05, while the other samples either did not change or increased by 2.5-fold (not significantly). The adhesion of NP-01-03 decreased to minus 1.85 pN, in comparison to other types of nanomaterials, in which it increased, including to positive values in NP-01-05. All differences were quite statistically reliable. It should be noted that there was a general tendency of materials to increase adhesion after their modifications, which may indicate an increase in their ability to accumulate on the surface of tumor and endothelial cells, as well as on the dense contacts between them [31,50]. There was a tendency for the Young’s modulus of the NPs to decrease when they were conjugated with FITC and to increase when exposed to other molecules. This may increase the probability of uptake of NPs by cells. All these factors, together with active targeting of NPs to GBM markers, may lead to rapid accumulation and uptake of NPs on the cell surface.

In a study of NP accumulation in a model blood vessel, it was found that NPs with larger sizes accumulate in the vessel more efficiently, as do nanomaterials with a positive surface charge. It was demonstrated in [51] that PLGA-based NPs having the same size as our NPs due to their negative charge could not pass the BBB. By increasing the chitosan content in the stoichiometric ratio of polymers in the initial mixtures, the NP content in the vessel increased. There was no clear correlation between the values of the nanomechanical properties of NPs and their accumulation in the BBB model. In addition, the differences in the vessel fluorescence data were not statistically significant, which may imply the importance of a large number of factors for BBB permeability at the same time. In article [20], the authors investigated the transmission of an hCMEC/D3 endothelial cell monolayer by chitosan- and butyl–chitosan-based NPs, which also had a positive surface charge and sizes of both 170 nm and 456 nm, and in all cases they tended to accumulate at dense contacts and further pass the BBB. This was explained by the importance of ZO-dependent paracellular transport, which carries various substances, including chitosan through dense contacts. The authors of the work [34] evaluated the accumulation of NPs based on aminated polystyrene with a size of 60 nm in HMEC endothelial cells. This type of NPs contained a large number of amino groups, like the materials obtained in our work. These materials were taken up by endothelial cells by caveolin- and LAMP1-dependent endocytosis and also accumulated in lysosomes individually. Particles had large sizes in our work, which may reduce the importance of the caveolin-dependent uptake pathway, which transports particles as small as 50–80 nm. A similar situation was observed in [30], which investigated Lipoid S 75-based NPs targeting cell-penetrating peptides and transferrin as octodecylamine delivery vehicles. HBMEC endothelial cells also absorb them predominantly by caveolin-dependent endocytosis, due to their size of up to 80 nm, as well as transferrin-mediated endocytosis due to active targeting. Also, the particles obtained in our work were clustered on blood vessel sections, which was confirmed by SEM and LSM with Rhodamine 6G and Hoechst 33342 dyeing, which also does not correlate with the peculiarities of the accumulation of NPs brought into the cell by LAMP1-dependent transcytosis. The authors of [52] investigated the interactions of gold-based NPs targeted by lectin and L-fucose with VE-Cadherin-positive HUVEC endotheliocytes located in a microfluidic vessel at a flow rate of 5 mL/min. Due to active targeting, particles accumulated on cells in approximately 24 h, followed by uptake by 36 h. Clustering of NPs with time was also observed. However, active targeting may not always lead to the desired result, as in [29], where a positive correlation between the accumulation and endocytosis of hCMEC/D3 cells of poly(N-isopropylmethacrylamide) NPs and a negative correlation with transcytosis when targeting NPs on transferrin was found. Based on the above literature data, we hypothesize that the nanomaterials obtained from our work are able to pass the BBB. The transfer is presumably strongly influenced by ZO-dependent paracytosis, but other transport pathways are also possible.

Both TMZ-loaded and non-loaded NPs significantly reduced survival of the U87-MG glioblastoma cell line. Unloaded ones inhibited cell growth by 30% in 1.5 days. In their work [53], the authors obtained chitosan- and fucoidan-based nanomaterials with sizes as small as 170 nm and a zeta-potential of 21 mV containing gemcitabine and an antibody to ErbB-2. These particles exhibited 85% cytotoxicity toward SKBR3 lines and 25% toward EA lines. Pure gemcitabine exhibited 15% and 30% cytotoxicity, respectively. This may be confirmation of the fact that, in addition to the chemotherapeutic agent, the NPs themselves also influence the proliferation of tumor cells. The effects of pectin and calcium chloride-based NPs with an average size of 120 nm and a surface charge of minus 8 mV on U87-MG cells were demonstrated in [54]. These materials had no effect on proliferation or increased the proliferation of glioblastoma cells to about 15% within 3 days. TMZ itself and the NPs containing it killed the cells almost immediately, which may suggest that the chemotherapeutic agent acts on the cells immediately and also exits the NPs during addition to the cells and kills the cells. The choice of chemotherapeutic agent also affects the effect on glioblastoma cells. Thus, hyaluronic acid and chitosan-based NPs targeting CD-44 in glioblastoma cells and containing curcumin, which reduces the survival of C6 glioblastoma cells to 75% by itself and to 55% in NPs within 24 h, were obtained in [19]. The particles were actively absorbed for 6 h, which was attributed to a diameter of 205 nm and a zeta-potential of 25 mV. While chitosan and hyaluronic acid-based nanomaterials with sizes up to 160 nm and a zeta-potential of minus 28 mV co-enriched with doxorubicin and cisplatin were obtained from [53]. These particles reduced cell survival of MCF-7 cell line to 8%, while pure chemopreparations together reduced cell survival to 25% in 3 days. The authors of article [55] described a method of TMZ delivery using NPs based on tripolyphosphate and chitosan with a size of up to 90 nm and a surface charge of 19 mV embedded in polyurethane nanofibers. The particles decreased the survival rate of glioblastoma cell line U251 from 67% on day 1 to 25% on day 7, compared to a decrease in survival rate after TMZ addition from 65% to 60%. However, unlike our work, the concentration administered to the NPs during sorption was not 4 mM in 0.4% pure DMSO solution, but 0.8 mM in acetone solution. In addition, it was not added to the NPs, but a conjugate of chitosan with TMZ was performed using Tween-80, after which the NPs in this work were centrifuged at a lower speed of 16,090× *g* for 20 min. These differences could account for the differences in the results. In addition to delivering low-molecular-weight chemotherapy drugs, NPs can also be effective carriers of other molecules that inhibit tumor cell growth and survival––ribosome-inactivating proteins [56] and small interfering RNAs [57] that aim to knock out the gene encoding galectin-1 in glioblastoma cells. Based on these findings, we hypothesize that pectin and chitosan-based NPs loaded with TMZ effectively inhibit the proliferation of U87-MG glioblastoma cells.

## 5. Conclusions

The aim of this work was to obtain nanoscale systems for immobilization of antitumor drugs and to confirm the possibility of their passage through the BBB. Nanoscale carbohydrate materials based on the oppositely charged macromolecules chitosan and pectin polysaccharides were created. Their surface modification with fluorescein for bioimaging, as well as antibodies specific to brain tumor cells—gliomas—was carried out. Correlations were made between the size, the surface charge, and the nanomechanical properties of the particles and their ability to immobilize TMZ, as a model drug. Modeling of the drug sorption process by NPs was carried out. Experiments on the cytotoxicity of NPs towards the tumor cell line U87-MG were carried out. A BBB system was modeled, and transfer of chitosan–pectin NPs through it was realized.

## Figures and Tables

**Table 1:** Compositions and abbreviations of the NPs.

Concentration Ratio of Pectin and Chitosan (wt.%)	0.1 wt.%/0.1 wt.%	0.1 wt.%/0.3 wt.%	0.1 wt.%/0.5 wt.%
	NPs-01-01	NPs-01-03	NPs-01-05
Additional surface molecules: FITC and antibodies (Ab) to NCAM-1 and PDL-1 receptors	NPs-01-01-FITC	NPs-01-03-FITC	NPs-01-05-FITC
	NPs-01-01-Ab	NPs-01-03-Ab	NPs-01-05-Ab
	NPs-01-01-FITC-Ab	NPs-01-03-FITC-Ab	NPs-01-05-FITC-Ab

**Table 2:** Drug loading over 24 h, during which the coefficient of correlation (R^2^) demonstrated behaviors of first-order and Higuchi kinetic models.

NP Type	First-Order			Higuchi	
	Slope	Half-Load Time min^−1^	R^2^	Slope	R^2^
NPs-01-01	0.098	7.07	0.885	5.05 × 10^−3^	0.988
NPs-01-03	0.127	5.45	0.950	6.78 × 10^−3^	0.969
NPs-01-05	0.184	3.77	0.889	10.78 × 10^−3^	0.997

**Table 3:** Kinetic equations for the TMZ loading process in the coordinates of the Higuchi and Nakai–Tachikawa models for pectin–chitosan NPs.

NP Type	Higuchi Model		Nakai–Tachikawa Model	
	Linear Equation	R^2^	Linear Equation	R^2^
NPs-01-01	(1 − f_t_)^⅓^ = 0.8043 − 0.0025 × t^1/2^	0.9813	f(Q_t_/Q_o_) = 0.0004 × t + 0.4873	0.9965
NPs-01-03	(1 − f_t_)^⅓^ = 0.7918 − 0.0037 × t^1/2^	0.9974	f(Q_t_/Q_o_) = 0.0024 × t + 0.4942	0.9920
NPs-01-05	(1 − f_t_)^⅓^ = 0.8220 − 0.0064 × t^1/2^	0.9918	f(Q_t_/Q_o_) = 0.0016 × t + 0.4849	0.9562

**Table 4:** Mathematics model studying diffusion for drug loading from medium into NPs of various ratios of pectin and chitosan.

NP Type	Loading Rate		Diffusion			Absorption/Diffusion Number
	K_r_ (10^−3^), min^−1^	R^2^	Mean Diameter, μm	D (10^−14^), cm^−2^/min	R^2^	ADN = K_r_. × r^2^/D
NPs-01-01	2.5	0.9918	0.128	0.38	0.9965	26.9
NPs-01-03	3.7	0.9813	0.169	3.99	0.9920	6.60
NPs-01-05	6.4	0.9974	0.196	3.58	0.9562	17.2

## Data Availability

Images and data are available from the corresponding author upon reasonable request. The data are not publicly available, due to their originality.

## Supplementary Materials

The following supporting information can be downloaded at: https://www.mdpi.com/article/10.3390/polym17121690/s1, Table S1: Different mechanisms of barrier passage by NPs.

## Abbreviations

The following abbreviations are used in this manuscript:

ADN	Absorption/diffusion number
AFM	Atomic force microscopy
BBB	Blood–brain barrier
DMSO	Dimethyl sulfoxide
EDC	1-[3-(Dimethylamino)propyl]-3-ethylcarbodiimide hydrochloride
FITC	Fluorescein isothiocyanate
FTIR	Fourier transmission infrared spectroscopy
GBM	Glioblastoma multiforme
HEPES	2-[4-(2-hydroxyethyl) piperazine-1-yl]-ethane-1-sulfonic acid
LSM	Laser scanning microscopy
NHS	N-hydroxysuccinimide
NPs	Nanoparticles
PBS	Phosphate-buffered saline
PEC	Polyelectrolyte complex
SEM	Scanning electron microscopy
TMZ	Temozolomide
UV	Ultraviolet-visible

## Appendix A

**Table A1:** Compositions and physicochemical parameters of the obtained chitosan–pectin PEC samples.

NP Composition	Median Size, nm (95% CI)	Average Size, nm ± SEM	Zeta-Potential, mV (Median, Interquartile Range)
Pectin 0.1% Chitosan 0.1% No modifications	55.55; 95% CI 46.52–64.83	128 ± 16.34	24.4 (IQR 107.0)
Pectin 0.1% Chitosan 0.1% FITC-labeled	104.4; 95% CI 99.31–110.8	128.1 ± 5.538	25.1 (IQR 117.3)
Pectin 0.1% Chitosan 0.1% Ab and FITC-conjugated	183.7; 95% CI 132.1–214.3	199 ± 17.4	−9.16 (IQR 112.6)
Pectin 0.1% Chitosan 0.3% No modifications	62.09; 95% CI 51.96–93.52	169.3 ± 24.86	28.2 (IQR 129.8)
Pectin 0.1% Chitosan 0.3% FITC-labeled	106.3; 95% CI 98.19–123.6	169 ± 31.95	32.9 (IQR 130.5)
Pectin 0.1% Chitosan 0.3% Ab and FITC-conjugated	177; 95% CI 133.6–287.9	206.2 ± 23.07	32.4 (IQR 115.4)
Pectin 0.1% Chitosan 0.5% No modifications	126.6; 95% CI 109–172.7	195.6 ± 16.72	31.2 (IQR 110.5)
Pectin 0.1% Chitosan 0.5% FITC-labeled	133.3; 95% CI 100.3–192.2	194.7 ± 22.35	36.7 (IQR 140.6)
Pectin 0.1% Chitosan 0.5% Ab and FITC-conjugated	208.7; 95% CI 180.6–241.3	228.5 ± 13.23	2.4 (IQR 110.6)

**Table A2:** Raman bands and their assignments (NPs-01-01 and modifications). Correlation of lines was carried out on the basis of [38,41,42,43].

Functional Group, Classification	NPs-01-01, cm^−1^	NPs-01-01 with FITC, cm^−1^	NPs-01-01 with Ab, cm^−1^	NPs-01-01 with FITC and Ab, cm^−1^
ν(O-H), water, and intramolecular H-bonds	3280 (s. *) 3225 (s.)	3271 (s.) 3216 (s.)	3274 (s.) 3215 (s.)	3274 (s.) 3214 (s.)
ν(N-H) in NH_3_	3191 (s.)	3191 (s.)	3191 (s.)	3191 (s.)
ν(C-H) in CH_3_	- -	2957 (m.) 2923 (m.)	- -	- -
ν(C-H) in CH_2_	-	2859 (m.)	-	-
ν(C=O) in COOH	1720 (m.)	1744 (m.)	1743 (m.)	1752 (m.)
ν(C=O) in acetylated monosaccharide residues	1670 (s.)	1662 (s.)	1680 (s.)	-
(1) δ(N-H) (2) νas(C=O) in ionized carboxyl (COO^−^) polysaccharides in the salt forms of pectins (3) xanthene C-C with conjugated carbonyl band (in FITC)	1600 (s.)	1605 (s.)	1591 (s.)	1626 (s.) 1591 (s.)
δ(N-H) in ionized amino (−NH_3_^+^)	1537 (s.)	1551 (s.)	1547 (s.)	1547 (s.)
in plane ring vibrations in FITC	-	-	-	1503 (s.)
δ(C-H) corresponds to C-H group symmetrical deformation due to the presence of saturated carbon atoms in the polysaccharide molecular structures	1437 (s.)	1442 (s.)	1431 (s.)	-
(1) δ(C-N) (2) δ(C-H) ring stretching vibrations (3) xanthene C-C stretch in FITC	1377 (s.) 1308 (m.)	1393 (s.) 1328 (s.)	1392 (s.) 1332 (s.)	1359 (s.)
phenoxide ion stretch conjugated with xanthene ring stretching in FITC	-	1296 (s.)	-	1359 (s.)
δ(O-H)	-	1234 (s.)	1220 (s.)	
ν(C-O-C) in glycosidic bond	-	1161 (m.)	-	1297 (s.)
ν(C-O-C) in pyranose ring structure	1100 (w.)	-	1118 (m.)	1236 (s.)
ν(C-O) asymmetric stretching in ring	-	1020 (m.)	1053 (m.)	1136 (m.)
ν(C-O-C) in glycosidic bond	941 (m.)	952 (m.)	938 (m.)	-
δ(C-H) corresponds to out-of-plane vibrations of glucosamine	879 (w.)	-	-	1074 (w.)
δ(C-H) corresponds to out-of-plane vibrations of glucosamine (~764 cm^−1^ − xanthene ring deformation in FITC)	800 (w.)	761 (m.)	793 (w.)	793 (w.) 727 (w.)
wagging vibrations of N-H and O-H	697 (w.) 556 (w.)	674 (w.)	633 (w.) 567 (m.)	640 (w.) 566 (m.
δ(C–O–C)	465 (w.)	460 (m.)	467 (m.)	-
δ(C-NH-C)	238 (m.)	241 (s.)	242 (s.)	238 (s.)

* Intensities: w.—weak, m.—medium, s.—strong.

**Table A3:** Raman bands and their assignments (NPs-01-03 and modifications). Correlation of lines was carried out on the basis of [38,41,42,43].

Functional Group, Classification	NPs-01-03, cm^−1^	NPs-01-03 with FITC, cm^−1^	NPs-01-03 with Ab, cm^−1^	NPs-01-03 with FITC and Ab, cm^−1^
ν(O-H), water, and intramolecular H-bonds	3278 (s. *) 3208 (s.)	3270 (s.) 3222 (s.)	3274 (s.) 3208 (s.)	3276 (s.) 3214 (s.)
ν(N-H) in NH_3_	3189 (s.)	3188 (s.) 3014 (m.)	3188 (s.) 3065 (s.)	3194 (s.) 3022 (m.)
ν(C-H) in CH_3_	2970 (m.)	3188 (s.) 3014 (m.)	2923 (w.)	3194 (s.) 3022 (m.)
ν(C-H) in CH_2_	-	2980 (m.) 2922 (m.)	2893 (w.)	-
ν(C=O) in COOH	1729 (m.)	1744 (m.)	1740 (m.)	1752 (m.)
ν(C=O) in acetylated monosaccharide residues	1679 (s.)	1662 (m.)	1680 (m.)	-
(1) δ(N-H) (2) νas(C=O) in ionized carboxyl (COO^−^) polysaccharides in the salt forms of pectins (3) xanthene C-C with conjugated carbonyl band (in FITC)	1627 (s.) 1600 (s.)	1600 (s.)	1656 (s.) 1598 (s.)	1595 (s.)
δ(N-H) in ionized amino (-NH_3_^+^)	1549 (s.)	1533 (s.)	1540 (s.)	1542 (s.)
δ(C-H) corresponds to C-H group symmetrical deformation due to the presence of saturated carbon atoms in the polysaccharide molecular structures	1471 (s.)	1440 (s.)	1435 (s.)	1435 (s.)
(4) δ(C-N) (5) δ(C-H) ring stretching vibrations (6) xanthene C-C stretch in FITC	1395 (s.) 1337 (s.)	1389 (s.) 1332 (s.)	1391 (s.) 1331 (s.)	1389 (s.) 1330 (s.)
phenoxide ion stretch conjugated with xanthene ring stretching in FITC	1300 (s.)	1300 (s.)	1300 (s.)	-
δ(O-H)	-	-	1208 (s.)	1220 (s.)
ν(C-O-C) in glycosidic bond	-	-	1120 (s.)	1163 (m.)
ν(C-O-C) in pyranose ring structure	-	1147 (w.)	1118 (s.)	1117 (s.)
ν(C-O) asymmetric stretching in ring	-	1021 (m.)	1043 (s.)	1049 (m.)
ν(C-O-C) in glycosidic bond	905 (w.)	932 (m.)	939 (s.)	956 (m.)
δ(C-H) corresponds to out-of-plane vibrations of glucosamine	843 (w.)	-	-	-
δ(C-H) corresponds to out-of-plane vibrations of glucosamine (~764 cm^−1^ − xanthene ring deformation in FITC)	-	764 (s.)	-	763 (m.)
wagging vibrations of N-H and O-H	638 (w.) 545 (w.)	667 (s.) 548 (s.)	639 (m.) 580 (s.)	657 (m.) 576 (s.)
δ(C–O–C)	456 (m.)	456 (m.)	468 (m.)	454 (m.)
γ(O-H)	386 (m.)	-	-	377 (m.)
δ(C-NH-C)	246 (s.)	245 (s.)	247 (s.)	247 (s.)

* Intensities: w.—weak, m.—medium, s.—strong.

**Table A4:** Raman bands and their assignments (NPs-01-05 and modifications). Correlation of lines was carried out on the basis of [38,41,42,43].

Functional Group, Classification	NPs-01-03, cm^−1^	NPs-01-03 with FITC, cm^−1^	NPs-01-03 with Ab, cm^−1^	NPs-01-03 with FITC and Ab, cm^−1^
ν(O-H), water, and intramolecular H-bonds	3272 (s. *) 3212 (s.)	3275 (s.) 3211 (s.)	3264 (s.) 3218 (s.)	3268 (s.) 3213 (s.)
ν(N-H) in NH_3_	3188 (s.) 3062 (m.) 3003 (w.)	3187 (s.) 3062 (m.) 3004 (w.)	3189 (s.) 3069 (s.)	3189 (s.)
ν(C-H) in CH_3_	2971 (w.) 2925 (w.)	2971 (w.) 2924 (w.)	2923 (w.)	2957 (m.) 2922 (m.)
ν(C-H) in CH_2_	2898 (w.) 2860 (w.)	2859 (w.)	-	2858 (m.)
ν(C=O) in COOH	1740 (m.)	1740 (m.)	1740 (m.)	1733 (m.)
(1) δ(N-H) (2) νas(C=O) in ionized carboxyl (COO^−^) polysaccharides in the salt forms of pectins (3) xanthene C-C with conjugated carbonyl band (in FITC)	1632 (s.)	1635 (s.)	1659 (s.) 1595 (s.)	1648 (s.) 1595 (s.)
δ(N-H) in ionized amino (−NH_3_^+^)	1548 (s.)	1549 (s.)	1542 (s.)	1543 (s.)
δ(C-H) corresponds to C-H group symmetrical deformation due to the presence of saturated carbon atoms in the polysaccharide molecular structures	1446 (s.)	1446 (s.)	1431 (m.)	1429 (s.)
(1) δ(C-N) (2) δ(C-H) ring stretching vibrations (3) xanthene C-C stretch in FITC	1403 (s.) 1333 (s.)	1404 (s.) 1334 (s.)	1397 (s.) 1334 (s.)	1393 (s.) 1333 (s.)
phenoxide ion stretch conjugated with xanthene ring stretching in FITC	1287 (s.)	1287 (s.)	1284 (s.)	1282 (s.)
δ(O-H)	-	-	1221 (s.)	1219 (s.)
ν(C-O-C) in glycosidic bond	-	1168 (w.)	-	-
ν(C-O-C) in pyranose ring structure	-	-	1124 (m.)	1123 (m.)
ν(C-O) asymmetric stretching in ring	-	-	1054 (w.)	1032 (m.)
ν(C-O-C) in glycosidic bond	-	-	954 (w.)	952 (w.)
δ(C-H) corresponds to out-of-plane vibrations of glucosamine (~764 cm^−1^ − xanthene ring deformation in FITC)	-	-	-	762 (m.)
wagging vibrations of N-H and O-H	-	-	579(s.)	670 (m.) 574 (s.)
δ(C–O–C)	454 (s.)	453 (s.)	452 (s.)	458 (s.)
γ(O-H)	385 (s.)	384 (s.)	397 (s.)	397 (m.)
δ(C-NH-C)	244 (s.)	244 (s.)	249 (s.)	245 (s.)

* Intensities: w.—weak, m.—medium, s.—strong.
