# Supplementary material for: Development of Carbohydrate Polyelectrolyte Nanoparticles for Use in Drug Delivery Systems that Cross the Blood–Brain Barrier to Treat Brain Tumors
Source: Polymers (Basel). 2025 Jun 18;17(12):1690. doi: 10.3390/polym17121690 (PMC12196646; doi:10.3390/polym17121690)
Supplement: Supplementary file 1 [file polymers-17-01690-s001.zip › polymers-3649081-supplementary.pdf]

Supplementary Materials

Table S1. Different mechanisms of barrier passage by NPs.

Cells type	Uptake and overcoming pathway	Description*	Article
Endothelial cells	Clathrin-mediated transcytosis	When NPs interact with this protein, clathrin-fringed vesicles are formed from sections of the plasma membrane. They carry out endocytosis of small soluble NSF attachment protein receptors. The interaction occurs with colloidal structures with a solid colloidal phase up to 200 nm in size. The size and maturation of vesicles is regulated by directly determining and controlling the curvature of the membrane. Protein is expressed in the endothelium of any tissue.	[29]
	Transferrin-dependent transcytosis	The transferrin receptor promotes the absorption of iron by cells and the passage of metalloproteins through endothelial cells. It can play the role of an intermediary for the accumulation of NPs targeted by an antibody or transferrin on the surface of endothelial cells, followed by their passage through the monolayer. Protein is expressed in the endothelium of various tissues.	[30] [31]
	Caveolin-mediated transcytosis	When NPs interact with this protein, caveol-bordered vesicles are formed from sections of the plasma membrane. The interaction occurs with colloidal structures with a liquid colloidal phase up to 50 nm in size and a negative surface charge. The CAVN2 protein regulates the morphology of caveoles, causing the curvature of the membranes inside them in a tissue-specific manner. It is mainly expressed in the endothelium of the lungs and adipose tissue.	[32]
	ZO-mediated paracellular transport	The dense junction restricts the movement of substances through the paracellular space and serves as a boundary between the apical and basolateral domains of the plasma membrane of endothelial cells, which differ in composition. Binds and engages PATJ in tight junctions where it assembles, positions, and maintains adhesive joints. When NPs with a positive surface charge accumulate on the surface of dense contacts, the ZO recognition domain is activated. This protein recognizes substances such as certain salts, cationic lipids, and chitosan. After that, this protein is replaced from dense contacts and the passing substances and structures follow a concentration gradient. The protein is expressed in all endothelial and epithelial cells.	[31]
	LYVE1-dependent transcytosis	A ligand is a specific transporter that moves between intracellular organelles and the plasma membrane. It binds to pericellular hyaluronic matrices deposited on the surface of leukocytes and promotes cell adhesion and migration through the lymphatic endothelium. Delivery systems can be targeted using	[33]

		hyaluronate. It is expressed mainly in the lymphatic endothelium.	
	LAMP-1-dependent transcytosis	Glycoprotein of the lysosomal membrane, which plays an important role in lysosome biogenesis, regulation of pH in lysosomes, autophagy and cholesterol homeostasis. It acts as an important regulator of the pH of the lysosome lumen, acidifying them to achieve optimal hydrolase activity. Targeting is possible with carbohydrates and Respiratory syncytial virus F protein. It is expressed in the endothelium of various tissues.	[34]
Astrocytes	Clathrin-mediated endocytosis	When NPs interact with this protein, clathrin-fringed vesicles are formed from sections of the plasma membrane. They carry out endocytosis of small soluble NSF attachment protein receptors. The interaction occurs with colloidal structures with a solid colloidal phase up to 200 nm in size. The size and maturation of vesicles is regulated by directly determining and controlling the curvature of the membrane. It is expressed in astrocytic tissue to a lesser extent than in endothelium.	[35]
	COP2-dependent endocytosis	When targeted, it acts as a cargo receptor on the luminal side to incorporate secretory cargo molecules into transport vesicles and participates in the formation of vesicle envelopes on the cytoplasmic side. It participates in the transportation of G-protein coupled receptors (GPCR). Regulates the exocytic transfer of F2RL1, OPRM1, and P2RY4 from Golgi cells to the plasma membrane, thus contributing to receptor resensitization. Together with SEC16A, it forms an organized framework that defines the exit sites of the endoplasmic reticulum (ERES), some specific areas of the endoplasmic reticulum, where COPII vesicles are formed (PubMed:17005010). In addition to its role in vesicle transport, it can also function as a leucine sensor that regulates TORC1 signaling and, more indirectly, cellular metabolism, growth, and survival.	[31]
Perycytes	COP1-dependent endocytosis	When targeted, the signaling pathways of light-induced transcription factors such as HI5, HYH, and LAF1 are inhibited. As a result, shell cells reduce hydrodynamic pressure and increase the permeability of endothelial cells and tight contacts for NPs. It is expressed in pericytes.	[31]
Glioblastoma	CD-44-dependent endocytosis	A transmembrane receptor that interacts through its ectodomain with extracellular matrix components such as hyaluronic acid (to a greater extent), collagen, growth factors, cytokines, or proteases, and serves as a platform for signaling by assembling protein complexes containing receptor kinases through its cytoplasmic domain. With these components, NPs can be targeted at CD-44. It is actively expressed in glioma cells.	[18]

	Clathrin-mediated endocytosis	When NPs interact with this protein, clathrin-fringed vesicles are formed from sections of the plasma membrane. They carry out endocytosis of small soluble NSF attachment protein receptors. The interaction occurs with colloidal structures with a solid colloidal phase up to 200 nm in size. The size and maturation of vesicles is regulated by directly determining and controlling the curvature of the membrane. It is expressed in glioma cells to a lesser extent than in endothelium.	[20]
	Integrin-dependent endocytosis	The protein ensures the adhesion of intraepithelial cells, including glioblastomas, to monolayers of epithelial cells. It can provide the transfer of delivery systems from endothelial cells directly to glioma cells via E-cadherin.	[23]<br>[30]
	NCAM-1-dependent endocytosis	NCAM-1, when overexpressed in glioma cells, plays an important role in neuronal and glial cell adhesion, and therefore can be used as a target when aimed at glioma cells	[24]
	PDL-1-dependent endocytosis	PD-L1 provides evasion of antitumor immunity due to increased content and activity in glioblastoma cells. This may enhance the accumulation of structures on the surface of glioblastoma cells and cause their further absorption into them.	[25]
	CD133-mediated endocytosis	Targeting NPs for CD133-dependent endocytosis which transports cholesterol and lipoproteins from the extracellular space into glioblastoma cells may also be an effective strategy for drug delivery to cells of this disease	[21]
	CD68-mediated endocytosis	CD68-mediated phagocytosis is also common in glioblastoma cells induced when NPs are modified with lectins	[22]

* Some information has been taking from the Uniprot and Human Protein Atlas.
